# Plasma urate concentration and risk of coronary heart disease: a Mendelian randomisation analysis

**DOI:** 10.1016/S2213-8587(15)00386-1

**Published:** 2016-04

**Authors:** Jon White, Reecha Sofat, Gibran Hemani, Tina Shah, Jorgen Engmann, Caroline Dale, Sonia Shah, Felix A Kruger, Claudia Giambartolomei, Daniel I Swerdlow, Tom Palmer, Stela McLachlan, Claudia Langenberg, Delilah Zabaneh, Ruth Lovering, Alana Cavadino, Barbara Jefferis, Chris Finan, Andrew Wong, Antoinette Amuzu, Ken Ong, Tom R Gaunt, Helen Warren, Teri-Louise Davies, Fotios Drenos, Jackie Cooper, Shah Ebrahim, Debbie A Lawlor, Philippa J Talmud, Steve E Humphries, Christine Power, Elina Hypponen, Marcus Richards, Rebecca Hardy, Diana Kuh, Nicholas Wareham, Yoav Ben-Shlomo, Ian N Day, Peter Whincup, Richard Morris, Mark W J Strachan, Jacqueline Price, Meena Kumari, Mika Kivimaki, Vincent Plagnol, John C Whittaker, International Consortium for Blood Pressure (ICBP), George Davey Smith, Frank Dudbridge, Juan P Casas, Michael V Holmes, Aroon D Hingorani

**Affiliations:** a Centre for Cardiovascular Genetics, British Heart Foundation Laboratories, Institute of Cardiovascular Science, University College London, London, UK; b Centre for Clinical Pharmacology, University College London, London, UK; c Centre for Population Health Sciences, University of Edinburgh, Edinburgh, UK; d Clinical Trial Service Unit & Epidemiological Studies Unit (CTSU), Nuffield Department of Population Health, University of Oxford, Oxford, UK; e Department of Clinical Pharmacology, William Harvey Research Institute, Barts and The London School of Medicine and Denstistry, Queen Mary University of London, London, UK; f Department of Epidemiology & Public Health, UCL Institute of Epidemiology & Health Care, University College London, London, UK; g Department of Non-Communicable Disease Epidemiology, London School of Hygiene & Tropical Medicine, London, UK; h Department of Primary Care & Population Health, University College London, Royal Free Campus, London, UK; i Population Health Research Institute, St George's, University of London, London, UK; j Department of Surgery and Center for Clinical Epidemiology and Biostatistics, Perelman School of Medicine, University of Pennsylvania, Philadelphia, PA, USA; k Genetics Division, Research and Development, GlaxoSmithKline, Harlow, UK; l Institute for Social and Economic Research, University of Essex, Colchester, UK; m Institute of Cardiovascular Science and Farr Institute, University College London, London, UK; n Metabolic Unit, Western General Hospital, Edinburgh, UK; o Division of Health Sciences, Warwick Medical School, University of Warwick, Coventry, UK; p Population, Policy and Practice, UCL Institute of Child Health, University College London, London, UnK; q MRC Epidemiology Unit, Institute of Metabolic Science, University of Cambridge, Cambridge, UK; r MRC Integrative Epidemiology Unit, School of Social and Community Medicine, University of Bristol, Bristol, UK; s MRC Unit for Lifelong Health and Ageing at UCL, London, UK; t NIHR Barts Cardiovascular Biomedical Research Unit, Queen Mary University of London, London, UK; u Queensland Brain Institute, University of Queensland, QLD, Australia; v School of Population Health and Sansom Institute of Health Research, University of South Australia, Adelaide, SA, Australia; w School of Social and Community Medicine, University of Bristol, Bristol, UK; x South Australian Health and Medical Research Institute, Adelaide, SA, Australia; y UCL Genetics Institute, University College, London, UK; z Wellcome Trust Sanger Institute, Hinxton, Cambridge, UK

## Abstract

**Background:**

Increased circulating plasma urate concentration is associated with an increased risk of coronary heart disease, but the extent of any causative effect of urate on risk of coronary heart disease is still unclear. In this study, we aimed to clarify any causal role of urate on coronary heart disease risk using Mendelian randomisation analysis.

**Methods:**

We first did a fixed-effects meta-analysis of the observational association of plasma urate and risk of coronary heart disease. We then used a conventional Mendelian randomisation approach to investigate the causal relevance using a genetic instrument based on 31 urate-associated single nucleotide polymorphisms (SNPs). To account for potential pleiotropic associations of certain SNPs with risk factors other than urate, we additionally did both a multivariable Mendelian randomisation analysis, in which the genetic associations of SNPs with systolic and diastolic blood pressure, HDL cholesterol, and triglycerides were included as covariates, and an Egger Mendelian randomisation (MR-Egger) analysis to estimate a causal effect accounting for unmeasured pleiotropy.

**Findings:**

In the meta-analysis of 17 prospective observational studies (166 486 individuals; 9784 coronary heart disease events) a 1 SD higher urate concentration was associated with an odds ratio (OR) for coronary heart disease of 1·07 (95% CI 1·04–1·10). The corresponding OR estimates from the conventional, multivariable adjusted, and Egger Mendelian randomisation analysis (58 studies; 198 598 individuals; 65 877 events) were 1·18 (95% CI 1·08–1·29), 1·10 (1·00–1·22), and 1·05 (0·92–1·20), respectively, per 1 SD increment in plasma urate.

**Interpretation:**

Conventional and multivariate Mendelian randomisation analysis implicates a causal role for urate in the development of coronary heart disease, but these estimates might be inflated by hidden pleiotropy. Egger Mendelian randomisation analysis, which accounts for pleiotropy but has less statistical power, suggests there might be no causal effect. These results might help investigators to determine the priority of trials of urate lowering for the prevention of coronary heart disease compared with other potential interventions.

**Funding:**

UK National Institute for Health Research, British Heart Foundation, and UK Medical Research Council.

## Introduction

Plasma urate is a circulating product of human purine metabolism synthesised from hypoxanthine and xanthine by the action of the enzyme xanthine oxidoreductase. With extreme increases in urate concentration, monosodium urate crystals are deposited in the joints, soft tissue, and renal parenchyma, causing acute inflammatory arthropathy (gout), gouty tophi, and nephropathy, respectively.^1^ Although the causal role of increased circulating urate concentrations in gout has been shown by Mendelian randomisation analysis^2^ (and urate lowering is the main treatment), the role of urate in coronary heart disease has been under debate since the 19th century.^3^

Patients with established coronary heart disease have increased concentrations of plasma urate compared with individuals free of the disease. Furthermore, increased plasma urate concentration is associated with increased risk of incident coronary heart disease.^4^

Beneficial and deleterious actions of urate on the cardiovascular system have been reported, making the role of urate in atherosclerosis unclear. Urate ions have potentially atheroprotective, free-radical-scavenging properties, and infusion of urate might correct endothelial dysfunction.^5^ However, proatherogenic effects of urate have also been described, including induction of cellular oxidative stress leading to attenuated nitric oxide bioavailability (linked to platelet and endothelial cell activation, and vascular smooth muscle proliferation).^6^

**Figure 1:**
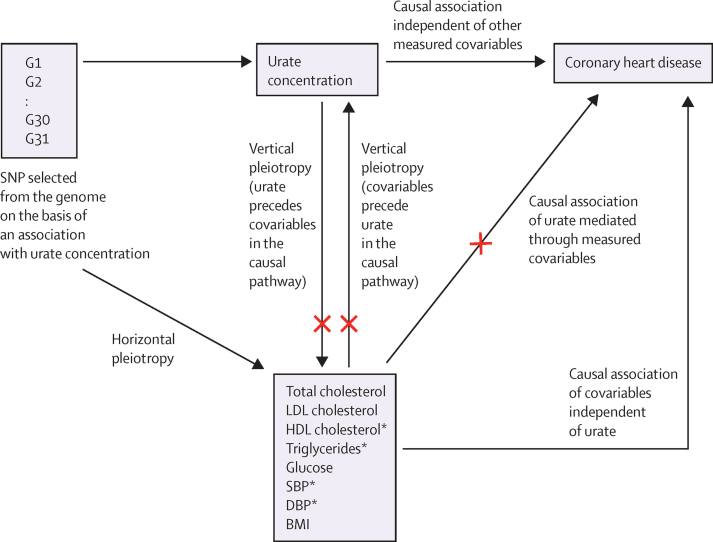
Conceptual framework for the Mendelian randomisation analysis of urate concentration and risk of coronary heart disease G1–31 are genes containing urate variants that together form the multilocus instrument for urate concentration. Horizontal pleiotropy occurs when the instrument associates with traits other than urate that become confounders if also associated with coronary heart disease. Vertical pleiotropy occurs if their level is affected by urate, and does not invalidate Mendelian randomisation analysis. SNP=single nucleotide polymorphism. SBP=systolic blood pressure. DBP=diastolic blood pressure. *Multivariable Mendelian randomisation, including DBP, SBP, HDL cholesterol, and triglycerides as covariates was used to account for possible horizontal pleiotropy arising from association of the instrument with these variables. The effect of the adjustment is to block the paths indicated with red crosses. Egger Mendelian randomisation analysis was used to account for unknown or unmeasured pleiotropic confounders.

In population studies, an increased urate concentration is associated with several risk factors for coronary heart disease, including high blood pressure, increased BMI, type 2 diabetes, reduced concentration of HDL cholesterol, and increased concentrations of triglycerides and LDL cholesterol.^4^ However, whether these variables confound or mediate the association of urate with coronary heart disease is uncertain (figure 1). Statistical adjustment for these variables in prospective observational studies attenuates the association of urate with coronary heart disease.^4^ Whether residual confounding results in over-estimation or whether the effect is underestimated because some of the variables are mediators remains unknown.

**Research in context**

**Evidence before this study**

The observational association between plasma urate and coronary heart disease is well established. However it remains in doubt whether this association is causal. Mendelian randomisation uses naturally occurring genetic variants that are allocated at random and associated with the risk factor of interest as an instrument to infer the causal role of a risk factor in a disease or outcome of interest. Previous Mendelian randomisation studies of plasma urate and risk of coronary heart disease have used single variants that affect plasma urate and reported discrepant findings.

**Added value of this study**

Using 31 independent single nucleotide polymorphisms (SNPs) identified as associated with plasma urate concentration from genome-wide association studies, we did a Mendelian randomisation analysis using three complementary approaches. Results from our conventional Mendelian randomisation analysis suggested that plasma urate might have a causal role in coronary heart disease; however, pleiotropic associations of the genetic instrument with several traits including blood pressure, triglycerides, and HDL cholesterol meant that the instrumental variable estimate from conventional Mendelian randomisation could be biased. Results from multivariate and Egger Mendelian randomisation analyses, which account for pleiotropy, both provided weaker evidence for a causal assoication of urate with coronary heart disease, with 95% CIs for both estimates including the null.

**Implications of all the available evidence**

Our findings suggest that the causal association, if any, between plasma urate and risk of coronary heart disease is likely to be modest. These data suggest that the observed association between plasma urate and coronary heart disease is probably affected by confounding by risk factors such as blood pressure and LDL cholesterol, HDL cholesterol, and triglycerides. Results of ongoing phase 3 randomised controlled trials will help to clarify this causal association, but any such trials could be underpowered if the predicted efficacy of the therapeutic modification of plasma urate has been based on effect estimates derived from existing observational data.

Results of randomised trials have provided some evidence that allopurinol (a urate-lowering drug) has beneficial effects on intermediate cardiovascular endpoints, including endothelial function, angina symptoms, blood pressure, left ventricular mass, and exercise capacity.^7^ Allopurinol acts through inhibition of xanthine oxidoreductase, which also reduces the generation of reactive oxygen species, which are formed as a byproduct of the metabolism of xanthine and hypoxanthine to urate.^8^, ^9^ Therefore, it remains unclear whether any benefits of allopurinol on these endpoints are due to urate lowering, inhibition of free-radical generation, or both. Moreover, no trial, with any urate-lowering drugs has yet reported an effect on clinically relevant cardiovascular endpoints,^10^ although a trial of this type is ongoing.

In this study, we estimated the extent of any causal relation between plasma urate concentration and risk of coronary heart disease using Mendelian randomisation.^11^ This type of analysis exploits the random allocation of genetic variants from parents to offspring at gametogenesis, protecting genotype-to-phenotype associations from the usual sources of confounding seen in observational studies and from reverse causation. Providing certain assumptions are met, if a genetic variant (or variants) associates with both a biomarker (eg, urate) and with risk of an outcome (coronary heart disease) in an instrumental variable regression, this would support a causal role for the biomarker in the outcome.^11^

Although Mendelian randomisation protects against many of the confounding factors that affect observational studies, it is potentially confounded by pleiotropy (the situation in which variation in a gene associates with multiple phenotypes). Pleiotropy can be vertical (wherein the gene affects more than one point in the same causal pathway) or horizontal (in which the gene affects more than one independent causal pathway). Whereas vertical pleiotropy does not necessarily breach the assumptions of Mendelian randomisation, unmeasured horizontal pleiotropy can lead to entirely spurious conclusions about causality.

Two methods have been proposed to address horizontal pleiotropy. The first simply includes the effect of the instrument on the pleiotropic factor as a covariate in the Mendelian randomisation analysis (termed multivariable Mendelian randomisation).^12^ The second uses Egger regression to account for the more general case in which there is a net pleiotropic effect on the instrument from multiple unmeasured sources (termed Egger Mendelian randomisation).^13^

We selected a set of single nucleotide polymorphisms (SNPs) identified from genome-wide association studies (GWAS) that were associated with urate concentration. Using these SNPs, we constructed a genetic instrument,^14^ and did conventional Mendelian randomisation (unadjusted for pleiotropy). Then, to account for pleiotropy, we used both multivariable Mendelian randomisation and Egger Mendelian randomisation.

Finally, we considered the genes and gene products tagged by these SNPs as potential therapeutic targets.

## Methods

### Study overview

We reviewed and updated the observational estimate for the association of plasma urate with risk of coronary heart disease and compared this with causal estimates from three different Mendelian randomisation approaches based on multiple plasma urate-associated genetic variants. We used summary estimates for effects of genotype on exposures and outcomes from the largest available meta-analyses of previous GWAS studies to address the research question, focusing on datasets with participants predominantly of European descent, to ensure allele frequencies were consistent over datasets and overcome possible modification of genetic effect by ancestral origin.

### Observational association between urate and coronary heart disease events and risk factors

We first did a fixed-effects meta-analysis of study summary estimates to update the meta-analysis of prospective observational studies by Wheeler and colleagues^4^ with the addition of 326 myocardial infarction or coronary revascularisation cases and 1618 controls from the British Women's Health and Heart Study (BWHHS), which was the only study available to the UCLEB Consortium^15^ with suitable data that had not already been included in the report by Wheeler and colleagues.^4^ This update gave a combined observational dataset of 17 studies, with 166 486 individuals and 9784 coronary heart disease events. Analyses were done without adjustment for renal function.

To estimate the observational association between urate and several coronary heart disease risk factors, including BMI, creatinine concentration, blood pressure, glucose concentration, HDL cholesterol, LDL cholesterol, total cholesterol, and triglycerides, we assimilated (by fixed-effects meta-analysis) data from UCLEB with studies that were included in the analysis by Wheeler and colleagues (appendix pp 1–2).^4^, ^15^

### Development of a genetic instrument for plasma urate concentration

To generate a genetic instrument for urate concentration, we searched for SNPs associated with urate concentration from the GWAS catalogue (accessed on Feb 18, 2015). We identified 31 independent loci (R^2^<0·3; separated by >140 kb) that had associations with urate at p<5 × 10^−7^ (appendix pp 1–2). Where the p value was greater than 5 × 10^−8^, inclusion was only on the basis of a clear functional role in urate metabolism (this applied to only one SNP, rs164009, that was previously designated to the gene *PRPSAP1* by the GRAIL [Gene Relationships Across Implicated Loci] process). In all cases, the SNP association had been replicated in studies done mainly in populations of European ancestry and effect sizes were taken from published meta-analyses. For each locus, we recorded the published effect size and the SE for the lead SNP (ie, the SNP with strongest association in the largest dataset). Where possible, we obtained effect size estimates for the lead SNP, or a suitable proxy, from additional publications (appendix pp 2–3) and combined the estimates for an SNP by fixed-effects meta-analysis. Details of lead SNPs and putative genes are reported in the appendix (p 3). Notably, an almost identical set of loci was used as an instrument for urate with a reported R^2^ of about 4·2% in the Rotterdam Study (n=5791).^16^ The 31 selected SNPs had been genotyped in the largest reported genetic association studies of coronary heart disease (CARDIoGRAMplusC4D, comprising C4D [Coronary Artery Disease consortium] and CARDIoGRAM [Coronary ARtery DIsease Genome wide Replication And Meta-analysis consortium]). Details of the original sources of information about SNP association with urate are provided in the appendix (pp 2–3). Genotyping in the UCLEB studies was done with the Illumina CardioMetabochip (Illumina, San Diego, CA, USA) and in the other consortia as described in the original publications.

We used a gene ontology enrichment analysis based on genes in closest proximity to the selected SNPs (AmiGO 2.1.4) to identify which gene ontology terms were over-represented in this set of genes relative to a null hypothesis that the SNPs were selected independently of their published associations (p values were obtained from the hypergeometric distribution).

### Instrumental variable analysis

The conventional instrumental variables linear regression analysis of the SNP effect on outcome versus the SNP effect on urate concentration was weighted by the inverse variance of the outcome effect estimate, and constrained (forced to pass through the origin). This approach equates to the summary method proposed by Johnson,^17^ and is the univariate case of the multivariate Mendelian randomisation method for summarised data described by Burgess and colleagues.^12^

To correct for observed pleiotropy, we included regression coefficients for phenotypes showing pleiotropy with the urate instrument as covariate in the instrumental variable model. Summary level association statistics used in the analysis were obtained from the relevant publications or from the public domain data deposits from the relevant GWAS (appendix pp 2–3), incorporating additional non-overlapping data from UCLEB where available.

Data for coronary artery disease or myocardial infarction contributed by CARDIoGRAMplusC4D investigators were downloaded from the CARDIoGRAMplusC4D website.

Summary statistics for the association of each of the 31 urate-associated SNPs with glucose, BMI, type 2 diabetes, plasma lipids, and blood pressure were obtained, respectively, from MAGIC (Meta-Analyses of Glucose and Insulin-related traits Consortium), GIANT (Genetic Investigation of ANthropometric Traits), DIAGRAM (DIAbetes Genetics Replication And Meta-analysis), GLGC (Global Lipids Genetic Consortium), and ICBP (International Consortium for Blood Pressure) GWAS consortia data (appendix p 2).

To test for unmeasured net pleiotropy, we used the Bowden and colleagues' method^13^ to test the hypothesis that the strength of the instrumental variable estimates of individual SNPs were symmetrically distributed around the point estimate. Symmetrical distribution suggests that pleiotropic effects, if present, are balanced and should not systematically bias the estimate of causal effect. To avoid the need to infer the SE, we resampled distributions of the summary statistics of the SNPs 100 000 times with replacement, recalculating the Mendelian randomisation estimate each time. We report statistical significance and CIs from this empirically derived distribution.

### Consistency between observational and instrumental variables analyses

We compared estimates for a 1 SD increase in urate generated with the instrumental variables meta-analysis with the updated observational estimate of the association between urate concentration and risk of coronary heart disease. Risk estimates of coronary heart disease in Wheeler and colleagues' study^4^ were originally reported as comparisons of the top versus bottom tertile of the urate distribution. To derive the per-SD estimate from this range, we exploited the properties of the normal distribution in which the top and bottom tertiles are separated by 2·18 SDs; we checked that the distribution of urate in participant data in the UCLEB Consortium data was approximately normal (appendix p 11).

### Sensitivity analyses

We examined the stability of the summary causal estimate by repeatedly (100 000 times) excluding six (∼20%) SNPs from the instrument, chosen at random in each cycle, and collecting the resulting instrumental variable estimates. By noting the proportion of these sensitivity coefficients that lie outside the CI from the normal distribution of the estimate with complete data, we obtained an indication of sensitivity. That is, when more than 5% of the sensitivity coefficients were outside the CI, there was evidence that the result was sensitive to SNP selection. We repeated the sensitivity analysis for an appropriate range of covariate models covering all phenotypes identified as potentially pleiotropic.

### Assessment of the proteins encoded by individual genes as therapeutic targets

To identify drugs and research compounds targeting proteins encoded by genes implicated by urate associated SNPs, we queried the ChEMBL (Chemical database of the European molecular biology laboratory) database (release chembl_19).^19^ To link genes to target identifiers in the ChEMBL database, we used the Ensembl Rest API and Uniprot web-services and thus obtained Uniprot accession keys representing the translated product of each gene queried.^19^, ^20^

Drugs were identified from the Mechanism/Binding Annotation table from ChEMBL release 19, which provides manually curated compound–target associations for licensed drugs. Research compounds (experimental drugs) were identified from the Activities table, which stores measured compound–target interactions. Results were limited to measurements from binding or functional assays with an assigned pChEMBL value, where pChEMBL is defined as −log10 (molar IC50, XC50, EC50, AC50, Ki, Kd or Potency). Assay targets were required to be identical or homologous to the submitted query.

### Statistical analysis

We estimated the power of the Mendelian randomisation analyses using Brion and colleagues' method.^21^ The origin and magnitude of the data used to generate the estimates are shown in the appendix (pp 4–5). For these calculations, we interpreted fully adjusted observational associations between urate concentration and cardiovascular risk factors and events as the most realistic approximation of the causal effect of urate. We also estimated power retrospectively using the instrumental variable estimates and corresponding SEs for each method. In this case, power was the complement of the false rejection rate, with a two-sided α of 0·05.

These prospective estimates of power suggested that we had 83% power to detect the same magnitude of association as for the observational association of urate concentration and risk of coronary heart disease (appendix p 4). However, with the instrumental variable estimates from the different methods in a retrospective analysis, we noted that available power to detect the effect of urate concentration on coronary heart disease was much lower than this (appendix pp 4, 5, 12) and that the Egger Mendelian randomisation analysis was notably lower in power than the other Mendelian randomisation methods used.

Data were analysed with R (version 2.15.2). Meta-analyses and Egger tests were done and forest and funnel plots were drawn with the metafor() package of R.

### Role of the funding source

The funders of the study had no role in the study design, data collection, data analysis, data interpretation, or writing of the report. JW had full access to all the data in the study and had final responsibility for the decision to submit for publication.

## Results

In our fixed-effects meta-analysis of prospective observational studies in which urate was quantified before incident coronary heart disease, plasma urate concentration was associated with increased risk of coronary heart disease: a 1 SD increased urate concentration was associated with an OR for coronary heart disease of 1·07 (95% CI 1·04–1·10; from 17 studies with 166 486 individuals and 9784 cases; *I*^2^ 41%; fixed-effects meta-analysis), after adjustment for age, sex, and other variables (appendix p 13). Urate concentration was also observationally associated with other established or putative risk factors for coronary heart disease, including age, smoking status, BMI, blood pressure, total cholesterol, and triglycerides (table 1).

Examination of the individual SNPs in the instrument in a meta-analysis of up to 68 studies, including 145 000 individuals, showed that each of the 31 SNPs selected for inclusion in the genetic instrument was associated with plasma urate concentration (appendix p 15). From a set of 21 804 annotated genes, those in proximity to the 31 urate-associated genetic variants showed significant functional enrichment for both urate and purine metabolism (appendix p 5).

We identified potential pleiotropic effects of a subset of the 31 SNPs. For example, in addition to associations with urate, SNPs within *OVOL1/LTBP3* and *ATXN2/PTPN11* were associated with systolic blood pressure, diastolic blood pressure, HDL cholesterol, and triglycerides; SNPs tagging *NFAT5, INHCB/INHCE, BAZ1B/MLX1PL*, and *GCKR* were associated with HDL cholesterol and triglycerides; SNPs within *TMEM171, IGFR1, SLC17A1/SLC17A3, ABCG2*, and *VEGFA* were associated with HDL cholesterol; and SNPs within *BCAS3* and *VEGFA* were associated with systolic blood pressure and diastolic blood pressure (appendix p 15 and p 17). We subsequently adjusted for this pleiotropy by the inclusion of combinations of these phenotype effect estimates as covariates in our multivariate Mendelian randomisation. Putative gene functions for these loci are noted in the appendix (pp 6–9).

In combination, the 31 SNP urate instrument was associated with systolic blood pressure, diastolic blood pressure, HDL cholesterol, and triglycerides (all p<0·05) (appendix pp 14–16), indicating pleiotropy of the instrument.

**Figure 2:**
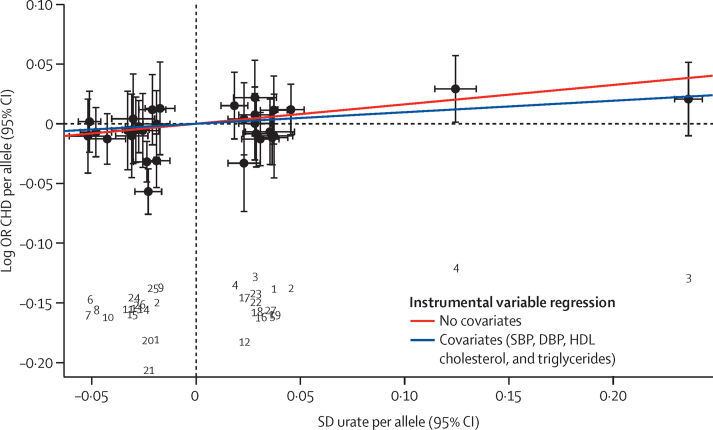
Association of individual SNPs with urate and coronary heart disease risk Estimates are derived from meta-analysis of data from several studies (appendix pp 2–3). Error bars represent 95% CIs. The numbers below the main figure correspond to the index column in the appendix (p 3) to allow cross-referencing. The slopes of the lines are instrumental variable regression estimates of the effect of urate on coronary heart disease risk with (blue) and without (red) SBP, DBP, HDL cholesterol, and triglycerides as covariates. OR=odds ratio. CHD=coronary heart disease. SNP=single nucleotide polymorphism. SBP=systolic blood pressure. DBP=diastolic blood pressure. HDL=high-density lipoprotein.

Data from roughly 145 000 individuals (68 studies) with information about genotype and urate concentration and 198 598 individuals (51 studies) with information about genotype and coronary heart disease (60 785 coronary heart disease events) were included in the Mendelian randomisation analysis of the association of plasma urate with risk of coronary heart disease. The instrumental variable effect estimate derived from conventional Mendelian randomisation was OR 1·18 (95% CI 1·08–1·29) per 1 SD increase in urate concentration (figure 2). The Cochran *Q* test showed heterogeneity among the instrumental variable estimates from individual SNPs (*Q*=47·67; p=0·02).

**Figure 3:**
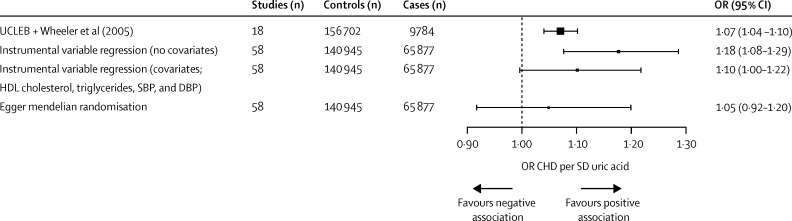
Observational and estimated causal association of plasma urate concentration risk of coronary heart disease Values represent a per 1 SD increase in urate concentration. Error bars represent 95% CIs. The vertical dotted line indicates the expectation under the null hypothesis (of no association between plasma urate and risk of coronary heart disease). SBP=systolic blood pressure. DBP=diastolic blood pressure. OR=odds ratio. UCLEB=University College London-London School of Hygiene & Tropical Medicine-Edinburgh-Bristol. HDL=high-density lipoprotein. CHD=coronary heart disease.

To examine the effect of the association of the 31 variants on systolic blood pressure, diastolic blood pressure, HDL cholesterol, and triglycerides on the Mendelian randomisation estimate, we included all combinations of systolic blood pressure, diastolic blood pressure, triglycerides, and HDL cholesterol as covariates in a multivariate Mendelian randomisation by including the genetic association of SNPs with these covariates in the instrumental variable regression analysis (figure 2). Multivariate Mendelian randomisation yielded an OR for risk of coronary heart disease of 1·10 (95% CI 1·00–1·22) per 1 SD difference in urate concentration (table 2; figure 3). It is important to note that multivariate Mendelian randomisation cannot account for pleiotropic influences that have not been measured.

**Figure 4:**
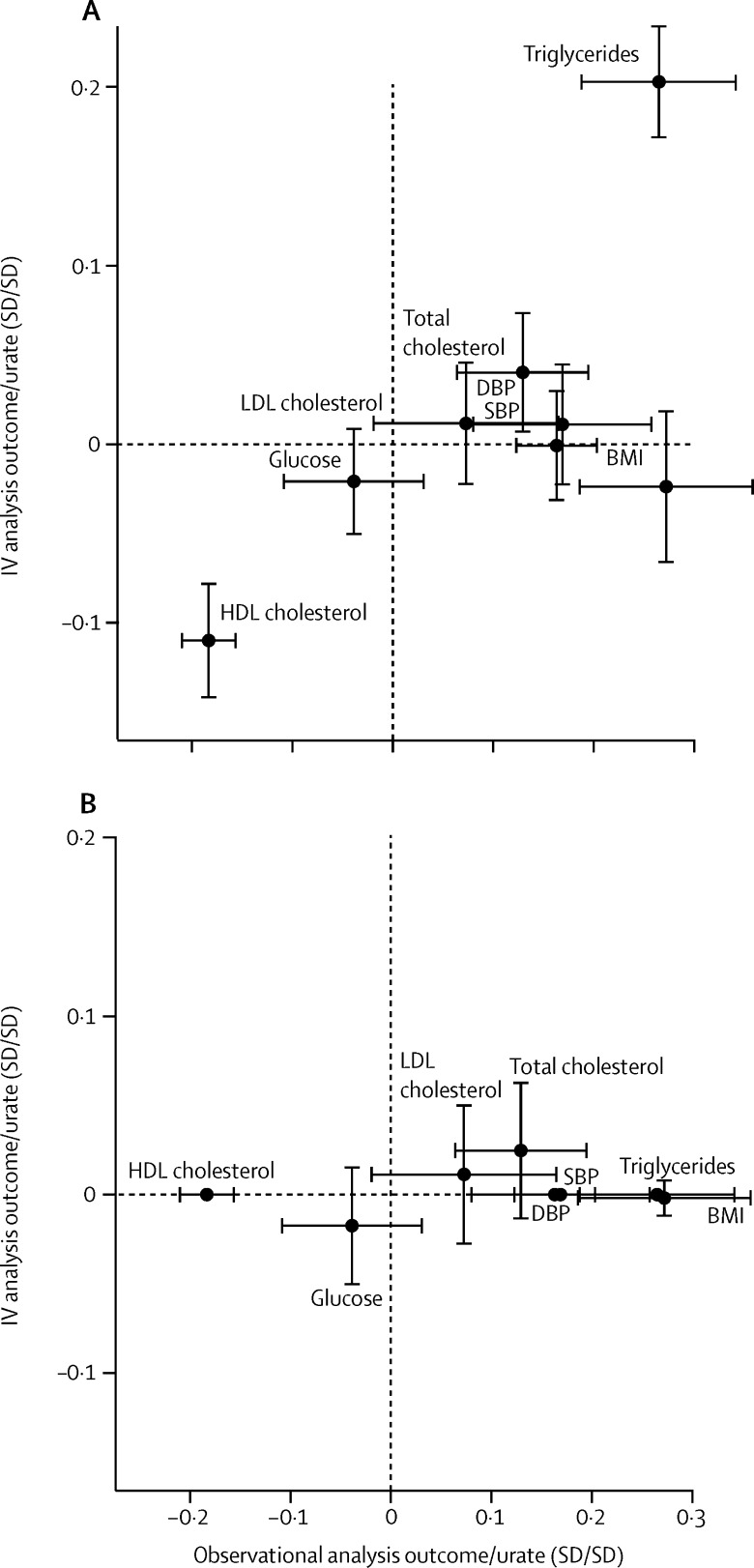
Comparison of observational and genetically instrumented associations between plasma urate concentration and several cardiovascular risk factors The genetically instrumented effect of urate without accounting for pleiotropic associations (A) and the genetically instrumented effect with DBP, SBP, HDL cholesterol, and triglycerides included as covariates in a multivariable Mendelian randomisation analysis (B). Error bars represent the 95% CIs. IV=instrumental variable. SBP=systolic blood pressure. DBP=diastolic blood pressure. HDL=high-density lipoprotein.

**Figure 5:**
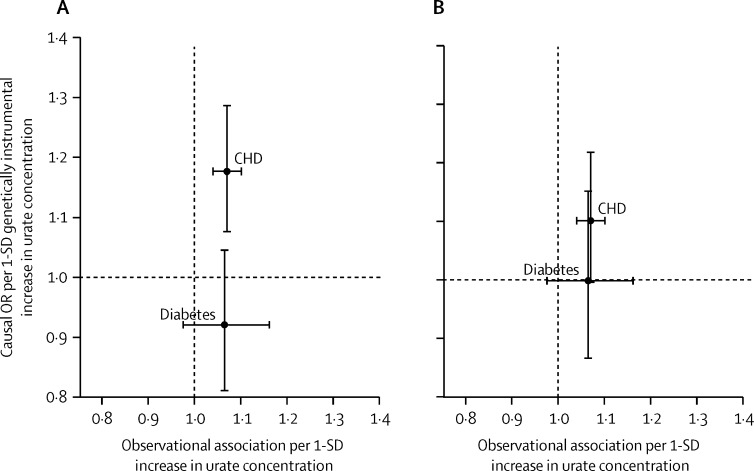
Observational association between binary traits and urate concentration against instrumental variable association for the 31 SNP instrument without covariates (A) and the 31 SNP instrument with DBP, SBP, HDL cholesterol, and triglycerides as covariates (B) Error bars represent 95% CI. OR=odds ratio. CHD=coronary heart disease. DBP=diastolic blood pressure. SBP=systolic blood pressure. HDL=high-density lipoprotein.

The Egger test suggested the presence of unmeasured pleiotropy of the instrument (Egger test p=0·01; appendix p 19). Using Egger Mendelian randomisation to account for this unmeasured pleiotropy, we derived a causal estimate of OR of 1·05 (95% CI 0·92–1·20) per 1 SD increase in urate (appendix p 19). This result underlines the potential for incomplete correction for pleiotropy obtained using multivariate Mendelian randomisation. Comparison of observational estimates to Mendelian randomisation estimates of the effect of urate concentration on all phenotypes studied are reported in figure 4 and figure 5.

We assessed the sensitivity of the Mendelian randomisation effect estimates to the exclusions of different combinations of six SNPs at random from the instrument and to the inclusion of different combinations of the covariates in a multivariable Mendelian randomisation analysis. Our results show that the model containing all covariates was not overly affected by SNP selection (appendix p 10). We noted that models containing combinations of systolic blood pressure, diastolic blood pressure, and HDL cholesterol seemed insensitive to SNP selection; however, the unadjusted (conventional Mendelian randomisation) model and the model with triglycerides alone gave higher effect size estimates and a larger proportion of those estimates were outside the 95% CI of the corresponding model fitted over effect estimates from all 31 SNPs. Egger Mendelian randomisation analysis proved insensitive to SNP selection, with only 3·8% of estimates lying outside the 95% CI for Egger regression estimates with all SNPs included.

One gene in the instrument (*SLC22A11*) encoded a target for probenecid, a drug previously used to lower urate concentration (appendix p 7). An instrumental variable analysis based solely on rs2078267 at the *SLC22A11* locus yielded an OR for risk of coronary heart disease of 1·19 (95% CI 0·75–1·78) per 1 SD increment in urate concentration. Another gene in the instrument (*VEGFA*) is targeted by monoclonal antibody therapeutics or an aptamer used to block angiogenesis in the treatment of certain cancers and age-related macular disease. Another (*IGF1R*) encodes the receptor for mecasermin (recombinant human insulin-like growth factor 1). The products of two other genes in the instrument (*ABGG2* and *GCKR*) were associated with compounds in the early stages of development.^22^

## Discussion

In this study, we investigated a potential causal role for plasma urate in the development of coronary heart disease using 31 SNPs identified from GWAS and using several complementary Mendelian randomisation approaches. The well powered, but potentially biased, conventional Mendelian randomisation analysis suggested a causal effect of urate on coronary heart disease. However, the 31 SNP genetic instrument showed pleiotropic associations with several cardiovascular risk factors (including systolic blood pressure and triglycerides) that could bias this effect estimate. Multivariate Mendelian randomisation regression analysis that adjusted for the associations of the genetic instrument with measured confounders yielded a causal estimate that was consistent with the results of both the meta-analysis of observational data and the conventional Mendelian randomisation analysis; however, the CIs for the causal effect derived from multivariate Mendelian randomisation were wider and included the null. Although multivariate Mendelian randomisation accounts for measured pleiotropy, as for conventional observational epidemiology, it cannot negate the effects of unmeasured or unknown confounding. Therefore, we used the recently developed Egger Mendelian randomisation analysis, which reduces inflation of a causal effect estimate due to both measured and unmeasured net pleiotropy, at the cost of lower power. Although this analysis confirmed the presence of unmeasured net pleiotropy, the causal estimate from Egger Mendelian Randomisation was again directionally consistent with the other two approaches, albeit with a smaller magnitude and even wider confidence limits (which, as for multivariate Mendelian randomisation, included the null). Taken together, the most conservative conclusion from these data is that plasma urate has a modest, if any, causal effect on risk of coronary heart disease.

The main assumptions of Mendelian randomisation are that the genetic variant strongly associates with the exposure, the genetic instrument associates exclusively with the risk factor of interest (and not with any confounders of the risk factor–disease outcome association); and the effect of the instrument on disease outcome is mediated exclusively through the risk factor of interest.^11^ In this study, we show a strong association of the genetic instrument with plasma urate, but we also show that our instrument was associated with potential confounders; however, we were able to deploy recently developed methods to account for this. Specifically, the genetic instrument showed association with HDL cholesterol, triglycerides, systolic blood pressure, and diastolic blood pressure, which could be due to horizontal or vertical pleiotropy between some of the SNPs included and these phenotypes. Although it is difficult to tease horizontal from vertical pleiotropy, that horizontal pleiotropy is the explanation for these findings is supported by the observation that the Mendelian randomisation association with risk of coronary heart disease generally persisted even after the associations of SNPs with systolic blood pressure, diastolic blood pressure, HDL cholesterol, and triglycerides were added to the model in multivariate Mendelian randomisation (appendix p 10).

Our finding adds to those from a previous Mendelian randomisation study of plasma urate concentration that included roughly 70 000 participants with more than 7000 cases of coronary heart disease from the Copenhagen General Population and Copenhagen City Heart Study.^23^ That study showed no evidence for a causal effect of urate on coronary heart disease.^23^ but it was based on a single urate-associated variant in *SLC2A9* (rs7442295), and, although the sample size was fairly large, it included only one-ninth of the number of coronary heart disease cases incorporated in the present analysis. Consistent with the Copenhagen study, in our much larger analysis, *SLC2A9* was not associated with coronary heart disease. Kleber and colleagues^24^ recently identified a causal effect of uric acid on cardiovascular death and sudden cardiac death in a dataset from 3315 patients admitted to hospital for angiography; however, this outcome is different from coronary heart disease. Two previous studies implicated a causal effect of urate on blood pressure: one that used only one SNP in *SLC2A9* (rs16890979)^25^ and another that used a 30 SNP score, but included only 5791 participants.^16^

Our study had several important strengths, but also some limitations. Strengths of the study include the incorporation of multiple urate-associated SNPs identified from GWAS to generate a genetic instrument with greater power than any single variant in isolation, the use of two-sample Mendelian randomisation methods that allow incorporation of summary effect estimates from very large, publicly available GWAS datasets (such as CARDIoGRAMplusC4D and DIAGRAM) to bolster power substantially, and the application of emerging approaches to Mendelian randomisation that allow statistical adjustment for measured confounders and adjustment for unbalanced net pleiotropy—(ie, multivariable and Egger Mendelian randomisation, respectively).

Limitations include much of the data coming from case-control studies from genetic discovery consortia, in which coronary heart disease cases are recruited after presentation with an acute coronary syndrome, which is contingent on survival. It is therefore possible that our findings were affected by survival advantage. However, the association of urate concentration with risk of coronary heart disease in prospective cohort studies (in which urate was measured before coronary heart disease events) provides evidence against survivorship bias. The mechanism by which some of the variants in our instrument affect urate concentration is not clear. However, understanding precise mechanisms is not a prerequisite for Mendelian randomisation, and seven of the 31 genes are known to be involved in the regulation of urate or purine metabolism. Finally, the observational association of plasma urate concentration with risk of coronary heart disease might be biased towards the null due to regression dilution bias, however absence of repeated measurements of plasma urate meant that it was not possible to assess the extent of this bias.^26^

Our study was designed to assess the causal role of plasma urate concentration in the risk of coronary heart disease, not the safety or efficacy of reducing plasma urate concentration through any particular therapeutic target. Randomised intervention trials will be necessary to test whether individual urate-lowering drugs might be effective for the prevention of coronary heart disease and have an acceptable safety profile. Allopurinol and febuxostat, which target xanthine oxidoreductase, as well as probenicid and sulfinpyrazone, which inhibit renal urate reabsorption, might be considered. Although variants in genes encoding drug targets of the probenecid and sulfinpyrazone were included in the genetic instrument (together with *GCKR,* which is a target for drug development for different reasons),^22^ in view of the imprecision around the causal estimates for individual SNPs (together with estimates derived from multivariate and Egger analyses), the efficacy (and safety) of these drugs for the prevention of coronary heart disease remains uncertain. Further genetic analyses focusing on SNPs in genes encoding the targets of urate-lowering drugs (eg, SNPs in *XDH*, which encoded xanthine oxidoreductase, the target of allopurinol, should these be shown to be associated with urate concentration), using a range of clinical outcomes, including but extending beyond coronary heart disease, would be necessary to address this distinct question, as has been done for other potential therapeutic targets.^27^, ^28^

Our study was also designed to inform on any potential causal role of plasma urate in the onset rather than the progression of or outcome from coronary heart disease. Different datasets would be needed to address the separate question of the effect of lowering plasma urate on outcome following a diagnosis of coronary heart disease, such as that assembled by the Genetics of Subsequent Coronary Heart Disease (GENIUS-CHD) consortium. We note, however, that a phase 3 randomised clinical trial of allopurinol (600 mg daily) plus standard care versus standard care alone in patients with established coronary heart disease designed to assess an effect on risk of coronary heart disease, stroke, and cardiovascular death is underway (ALL-HEART; UK Clinical Research Network UKCRN ID: 15328, Integrated Research Application System IRAS ID: 32017426).

In summary, genetic evidence based on conventional and novel Mendelian randomisation approaches suggest a modest, if any, causal effect of plasma urate concentration in the development of coronary heart disease. The findings might help investigators to judge the relative priority of plasma urate, as against other risk factors, as a therapeutic target for the prevention of coronary heart disease.

## Figures and Tables

**Table 1:** Observational associations of plasma urate concentration with cardiovascular risk factors

	Studies (n)*	**N or n:n**	**Difference in risk factor for a 1 SD higher plasma urate (95% CI)**	**p value**
**Continuous variables**				
HDL cholesterol (mmol/L)	4	22 669	−0·08 (−0·087 to −0·065)	<0·0001
LDL cholesterol (mmol/L)	2	19 195	0·07 (−0·019 to 0·163)	0·121
Total cholesterol (mmol/L)	5	68 446	0·14 (0·07 to 0·213)	0·0001
Triglycerides (mmol/L)	3	25 606	0·31 (0·216 to 0·393)	<0·0001
Fasting glucose (mmol/L)	3	14 571	−0·08 (−0·23 to 0·066)	0·276
Creatinine (mg/L)	2	6696	4·43 (1·235 to 7·634)	0·0066
BMI (kg/m^2^)	7	84 419	1·29 (0·879 to 1·694)	<0·0001
SBP (mm Hg)	7	84 419	3·31 (2·498 to 4·128)	<0·0001
DBP (mm Hg)	4	19 033	1·95 (0·926 to 2·977)	0·0002
Age (years)	3	5713	0·21 (0·045 to 0·383)	0·013
eGFR (mL/min/1·73m^2^)	2	4393	−4·59 (−4·905 to −4·269)	<0·0001
**Binary traits**				
Sex (female *vs* male)	3	3738:1975	0·80 (0·746 to 0·865)	<0·0001
Smoking (ever *vs* never)	2	2678:1615	1·11 (1·041 to 1·185)	0·0015
Diabetes (present *vs* absent)	2	517:3877	1·07 (0·976 to 1·162)	0·157

SBP=systolic blood pressure. DBP=diastolic blood pressure. eGFR=estimated glomerular filtration rate.

* Sources of data are reported in the appendix (p 2).

**Table 2:** Causal analysis of urate on risk of coronary heart disease derived from Mendelian randomisation analyses

	**Studies**	**N (cases)**	**31 SNP instrument**	Multivariate regression estimate*	**Egger Mendelian randomisation**
Coronary heart disease	58	206 822 (65 877)	1·1766 (1·0763 to 1·2861)	1·1013 (0·996 to 1·2178)	1·0488 (0·9191 to 1·1968)

Data are odds ratios per SD difference in plasma urate concentration (95% CI).

* Covariates are diastolic blood pressure, systolic blood pressure, trigycerides, and high-density lipoprotein. SNP=single nucleotide polymorphisms.

## References

[1] Terkeltaub RA (2010). Update on gout: new therapeutic strategies and options. Nat Rev Rheumatol.

[2] Yang Q, Köttgen A, Dehghan A (2010). Multiple genetic loci influence serum urate and their relationship with gout and cardiovascular disease risk factors. Circ Cardiovasc Genet.

[3] Davis N (1897). The cardiovascular and renal relations and manifestations of gout. JAMA.

[4] Wheeler JG, Juzwishin DM, Eriksdottir G, Gudnason V, Danesh J (2005). Serum uric acid and coronary heart disease in 9,458 incident cases and 155,084 controls: prospective study and meta-Analysis. PLoS Med.

[5] Waring WS, McKnight JA, Webb DJ, Maxwell SRJ (2006). Uric acid restores endothelial function in patients with type 1 diabetes and regular smokers. Diabetes.

[6] Cory DB, Eslami P, Yamamoto K, Nyby MD, Makino H, Tuck ML (2008). Uric acid stimulates vascular smooth muscle cell proliferation and oxidative stress via the vascular renin-angiotensin system. J Hypertens.

[7] Rajendra NS, Ireland S, George J, Belch JJF, Lang CC, Struthers AD (2011). Mechanistic insights into the therapeutic use of high-dose allopurinol in angina pectoris. J Am Coll Cardiol.

[8] Landmesser U, Drexler H (2002). Allopurinol and endothelial function in heart failure: future or fantasy?. Circulation.

[9] Struthers A, Shearer F (2012). Allopurinol: novel indications in cardiovascular disease. Heart.

[10] Stone PH (2001). Allopurinol: a new anti-ischemic role for an old drug. JACC.

[11] Lawlor DA, Harbrod RM, Sterne JAC, Timpson N, Davey Smith G (2008). Mendelian randomisation: using genes as instruments for making causal inferences in epidemiology. Stat Med.

[12] Burgess S, Dudbridge F, Thompson SG (2015). Multivariable Mendelian randomization: the use of pleiotropic genetic variants to estimate causal effects. Am J Epidemiol.

[13] Bowden J, Davey Smith G, Burgess S (2015). Mendelian randomization with invalid instruments: effect estimation and bias detection through Egger regression. Int J Epidemiol.

[14] Palmer TM, Lawlor DA, Harbord RM (2011). Using multiple genetic variants as instrumental variables for modifiable risk factors. Stat Met Med Res.

[15] Shah T, Engmann J, Dale C (2013). Population genomics of cardiometabolic traits: design of the University College London–London School of Hygiene and Tropical Medicine–Edinburgh–Bristol (UCLEB) Consortium. PLoS One.

[16] Sedaghat S, Pazoki R, Uitterlinden AG (2014). Association of uric acid genetic risk score with blood pressure: the Rotterdam study. Hypertension.

[17] Johnson T Efficient calculation for multi-SNP genetic risk scores. American Society of Human Genetics Annual Meeting; San Francisco, CA, USA; Nov 6–10, 2012. http://cran.r-project.org/web/packages/gtx/vignettes/ashg2012.pdf.

[19] Yates A, Beal K, Keenan S (2015). The Ensembl REST API: Ensembl data for any language. Bioinformatics.

[20] Jain E, Bairoch A, Duvaud S (2009). Infrastructure for the life sciences: design and implementation of the UniProt website. BMC Bioinformatics.

[21] Brion MJ, Shakhbazov K, Visscher PM (2013). Calculating statistical power in Mendelian randomization studies. Int J Epidemiol.

[22] Ashton KS, Andrews KL, Bryan MC (2014). Small molecule disruptors of the glucokinase–glucokinase regulatory protein interaction: 1. discovery of a novel tool compound for in vivo proof-of-concept. J Med Chem.

[23] Palmer TM, Nordestgaard BG, Benn M (2013). Association of plasma uric acid with ischaemic heart disease and blood pressure: mendelian randomisation analysis of two large cohorts. BMJ.

[24] Kleber ME, Delgado G, Grammer TB (2015). Uric acid and cardiovascular events: a Mendelian randomisation study. J Am Soc Nephrol.

[25] Parsa A, Brown E, Weir MR, Fink JC, Shuldiner AR, Mitchell BD, McArdle PF (2012). Genotype-based changes in serum uric acid affect blood pressure. Kidney Int.

[26] Clarke R, Shipley M, Lewington S, Youngman L, Collins R, Marmot M, Peto R (1999). Underestimation of risk associations due to regression dilution in long-term follow-up of prospective studies. Am J Epidemiol.

[27] Interleukin-6 Receptor Mendelian Randomisation Analysis (IL6R MR) Consortium (2012). The interleukin-6 receptor as a target for prevention of coronary heart disease: a mendelian randomisation analysis. Lancet.

[28] Holmes MV, Simon T, Exeter HJ (2013). Secretory phospholipase A_2_-IIA and cardiovascular disease: a Mendelian randomization study. J Am Coll Cardiol.

