# Supplementary material for: Plasma urate concentration and risk of coronary heart disease: a Mendelian randomisation analysis
Source: Lancet Diabetes Endocrinol. 2016 Apr;4(4):327–36. doi: 10.1016/S2213-8587(15)00386-1 (PMC4805857; doi:10.1016/S2213-8587(15)00386-1)
Supplement: Supplementary appendix [file mmc1.pdf]

THE LANCET
Diabetes & Endocrinology

Supplementary appendix

This appendix formed part of the original submission and has been peer reviewed. We post it as supplied by the authors.

Supplement to: White J, Sofat R, Hemani G, et al, for the UCLEB (University College London-London School of Hygiene & Tropical Medicine-Edinburgh-Bristol Consortium. Plasma urate concentration and risk of coronary heart disease: a Mendelian randomisation analysis. *Lancet Diabetes Endocrinol* 2016; published online Jan 15. http://dx.doi.org/10.1016/S2213-8587(15)00386-1.

<u>**Supplementary Material**</u>

Plasma urate and coronary heart disease: Mendelian randomisation analysis.

Jon White§, Reecha Sofat, Gibran Hemani, Tina Shah, Jorgen Engmann, Caroline Dale, Sonia Shah, Felix A Kruger, Claudia Giambartolomei, Daniel I. Swerdlow, Tom Palmer, Stela McLachlan, Claudia Langenberg, Delilah Zabaneh, Ruth Lovering, Alana Cavadino, Barbara Jefferis, Chris Finan, Andrew Wong, Antoinette Amuzu, Ken Ong, Tom R. Gaunt, Helen Warren, Teri-Louise Davies, Fotios Drenos, Jackie Cooper, Shah Ebrahim, Debbie A. Lawlor, Philippa J. Talmud, Steve E. Humphries, Christine Power, Elina Hypponen, Marcus Richards, Rebecca Hardy, Diana Kuh, Nicholas Wareham, Yoav Ben-Shlomo, Ian N. Day, Peter Whincup, Richard Morris, Mark W. J. Strachan, Jacqueline Price, Meena Kumari, Mika Kivimaki, Vincent Plagnol, John C. Whittaker, International Consortium for Blood Pressure (ICBP)[a], George Davey Smith, Frank Dudbridge, Juan P. Casas, Michael V. Holmes*§, Aroon D. Hingorani*, on behalf of the University College-London-School-Edinburgh-Bristol (UCLEB) Consortium.

* Contributed equally

§ Corresponding authors

[a] See: Supplementary Appendix 1 for list of International Consortium for Blood Pressure (ICBP) authors.

Contents

Supplementary Tables

Table S1 Sources of data for estimate of observational associations with plasma urate concentration. 1

Table S2. Sources of data used in this study. 2

Table S3. SNPs used to construct the genetic instrument for plasma urate. 3

Table S4. Association of the 31 SNP plasma urate instrument with cardiovascular traits. 4

Table S5. Proxy SNPs used in the Instrument. 4

Table S6. Power (two-sided α=0·05) for conventional IV regression of the binary outcomes. 4

Table S7. Power (α=0·05) for conventional IV regression of the continuous outcomes. 5

Table S8. Gene Ontology Enrichment Analysis. 5

Table S9. Function and druggability of genes represented in the genetic instrument for plasma urate. 6-9

Table S10. Sensitivity tests with different covariate models. 10

Supplementary Figures

Figure S1. The distribution of plasma urate concentration in the UCLEB consortium data. 11

Figure S2. Power curves derived from analytical outcomes. 12

Figure S3. Observational association between plasma urate concentration and relative risk of CHD in 17 prospective population-based cohorts. 13

Figure S4. The association of individual SNPs and the 31 SNP instrument for plasma urate concentration with continuous phenotypes. 14

Figure S5. The association of individual SNPs and the 31 SNP instrument for plasma urate concentration with continuous phenotypes. 15

Figure S6. The association of the individual SNPs and the 31 SNP instrument for plasma urate concentration with blood pressure 16

Figure S7. The association of the individual SNPs and the 31 SNP instrument for plasma urate concentration with binary phenotypes. 16

Figure S8. Funnel plot of individual IV beta estimates for SNPs in the instrument. 17

Figure S9. Sensitivity tests 18

Figure S10. Association of SNPs with plasma urate concentration and risk of CHD 19

Supplementary Appendix 1. Contributors to the ICBP 20

Supplementary References 21-22

Table S1. Sources of data for estimate of observational associations with plasma urate concentration.

Data Source	SBP	DBP	TC	TG	HDL-C	LDL-C	Creatinine	BMI	Glucose
Liese et al.1999.[s1]	1005	1005	1005	-	1005	-	1005	1005	-
Puddu et al. 2001.[s2]	2469	-	-	-	2469	-	-	2469	2469
Fang et al. 2000.[s3]	5926	5926	5926	-	-	-	-	5926	-
Medalie et al. 1973.[s4]	6411	6411	6411	6411	-	-	-	6411	6411
Moriarity et al. 2000.[s5]	13504	-	-	13504	13504	13504	-	13504	-
UCLEB consortium[s6]	5691	5691	5691	5691	5691	5691	5691	5691	5691
Tomita et al.2000.[s7]	49413	-	49413	-	-	-	-	49413	-

Note: Estimates reported for smoking, age, sex, eGFR and, diabetes were made in UCLEB consortium data only.

Table S2. Sources of data used in this study.

Association (A with B)	Data	N individuals	N studies
**Observational Association**			
Urate – lipid phenotypes	Leise *et al.* [s1]<br>Puddu *et al.* [s2]<br>Medalie *et al.* [s4]<br>Moriarty *et al.* [s5]<br>UCLEB [s6]	1 005<br>2 469<br>6 411<br>13 504<br>5 691	1<br>1<br>1<br>1<br>2*
Urate – BMI	Leise *et al.* [s1]<br>Puddu *et al.*[s2]<br>Medalie *et al.* [s4]<br>Moriarty *et al.* [s5]<br>Fang *et al.* [s3]<br>Tomita *et al.*[s7]<br>UCLEB[s6]	1 005<br>2 469<br>6 411<br>13 504<br>5 926<br>49 413<br>5 691	1<br>1<br>1<br>1<br>1<br>1<br>3§
Urate – T2D	UCLEB[s6]	4394	2*
Urate – fasting glucose	Leise *et al.* [s1]<br>Puddu *et al.* [s2]<br>Moriarty *et al.* [s5]<br>UCLEB [s6]	1 005<br>2 469<br>13 504<br>5 691	1<br>1<br>1<br>3§
Urate - BP	Leise *et al.* [s1]<br>Puddu *et al.* [s2]<br>Medalie *et al.* [s4]<br>Moriarty *et al.* [s5]<br>Fang *et al.* [s3]<br>Tomita *et al.*[s7]<br>UCLEB [s6]	1 005<br>2 469<br>6 411<br>13 504<br>5 926<br>49 413<br>5 691	1<br>1<br>1<br>1<br>1<br>1<br>3§
Urate - CHD	Wheeler *et al.*[4]<br>UCLEB [s6]	174 326(9 458 cases)<br>1 944 (326 cases)	17<br>1
**Genetic Association**			
SNP - urate	Köttgen *et al.*[s8]<br>Kolz *et al.* [s9]<br>UCLEB [s6]	110 347<br>27 817<br>7 151	48<br>14<br>3
SNP – CHD	CARDIoGRAM[s10] **and/or**<br>C4D[s11] **or**<br>CARDIoGRAM plus C4D[s12]<br>**with** UCLEB [s6]	78 856 (19 368 cases)<br>30 393 (15 357 cases)<br>186 203 (60 785 cases)<br>12 395 (2 131 cases)	37<br>14<br>48<br>7
SNP – lipid phenotypes	GLGC[s13]<br>UCLEB [s6]	187 190<br>9 431	64<br>4
SNP – T2D	DIAGRAM[s14]<br>UCLEB [s6]	69 033 (12 717 cases)<br>15 605 (2 643 cases)	12<br>8
SNP – fasting glucose	MAGIC [s15]<br>UCLEB [s6]	46 186<br>11 211	21<br>7
SNP - BP	ICBP [s16]<br>UCLEB [s6]	69 590<br>20 077	29<br>8
SNP - BMI	GIANT[s17]	127 600	64
**Confounding associations**			
TC - CHD	Liese et al, UCLEB, Fang *et al.* Tomita et al, Medalie *et al.*[s1,s3,s4,s6,s7]	64 446	5
HDLc - CHD	Liese et al, UCLEB, Puddu *et al.*, Moriarity *et al.*[s1,s2,s5,s6]	22 669	4
LDLc - CHD	UCLEB, Moriarity *et al.* [s5, s6]	19 195	2
TG - CHD	Moriarity et al, UCLEB, Medalie *et al.*[s4,s5, s6]	25 606	3
BMI - CHD	Fang et al, Moriarity *et al.*, Puddu *et al.*, Liese *et al.*, UCLEB, Tomita *et al.*, Medalie *et al.*[s1-s7,]	84 419	7
Fasting glucose - CHD	Puddu et al., UCLEB, Medalie et al. [s2,s4,s6]	15 471	3
SBP - CHD	Fang *et al.*, Moriarity *et al.*, Puddu *et al.*, Liese *et al.*, UCLEB, Tomita *et al.* Medalie *et al.*[s1,s4,s6,s7]	84 419	7
DPB - CHD	Liese *et al.*, UCLEB, Fang *et al.* Tomita *et al.*, Medalie *et al.* [s1,s3,s4,s6,s7]	19 033	4

* BRHS and BWHHS; § BRHS, BWHHS and CaPS; Moriarty et al consisted of 4 communities

Table S3. SNPs used to construct the genetic instrument for plasma urate[a].

Index	SNP	CHR	BP	GENE (nearest/GRAIL)	Allele	Meta-analysis beta	Meta-analysis SE	N	S	Source Data
1	rs1471633	1	144435096	PDZK1/PDZK1	A	0·0568	0·0050	116404	54	Köttgen[s8] and UCLEB[s6]
2	rs1260326	2	27584444	GCKR/GCKR	T	0·0693	0·0049	117293	54	Köttgen[s8] and UCLEB[s6]
3	rs12498742	4	9553150	SLC2A9/SLC2A9	A	0·3600	0·0051	145110	68	Köttgen[s8], UCLEB[s6] Kolz[s9]
4	rs2231142	4	89271347	ABCG2/ABCG2	T	0·1896	0·0077	140915	68	Köttgen[s8], UCLEB[s6] and Kolz[s9]
5	rs675209	6	7047083	RREB1/RREB1	T	0·0556	0·0059	117293	54	Köttgen[s8] and UCLEB[s6]
6	rs1165151	6	25929595	SLC17A1/SLC17A3	T	-0·0779	0·0042	145201	68	Köttgen[s8], UCLEB[s6] and Kolz[s9]
7	rs1171614	10	61139544	SLC16A9/SLC16A9	T	-0·0790	0·0070	110000	49	Köttgen[s8]
8	rs2078267	11	64090690	SLC22A11/SLC22A11	T	-0·0732	0·0058	117293	54	Köttgen[s8] and UCLEB[s6]
9	rs478607	11	64234639	NRXN2/SLC22A12	A	-0·0264	0·0056	137967	49	Köttgen[s8]
10	rs3741414	12	56130316	INHBC/INHBE	T	-0·0649	0·0068	117293	54	Köttgen[s8] and UCLEB[s6]
11	rs11264341	1	153418117	TRIM46/PKLR	T	-0·0500	0·0060	110000	49	Köttgen[s8]
12	rs17050272	2	121022910	INHBB/INHBB	A	0·0350	0·0060	110000	49	Köttgen[s8]
13	rs6770152	3	53075254	SFMBT1/MUSTN1	T	-0·0440	0·0050	110000	49	Köttgen[s8]
14	rs17632159	5	72467238	TMEM171/TMEM171	C	-0·0390	0·0060	110000	49	Köttgen[s8]
15	rs729761	6	43912549	VEGFA/VEGFA	T	-0·0470	0·0060	110000	49	Köttgen[s8]
16	rs1178977	7	72494985	BAZ1B/MLXIPL	A	0·0470	0·0070	110000	49	Köttgen[s8]
17	rs10480300	7	151036938	PRKAG2/PRKAG2	T	0·0350	0·0060	110000	49	Köttgen[s8]
18	rs2941484	8	76641323	HNF4G/HNF4G	T	0·0440	0·0050	110000	49	Köttgen[s8]
19	rs10821905	10	52316099	A1CF/ASAH2	A	0·0570	0·0070	110000	49	Köttgen[s8]
20	rs642803	11	65317196	OVOL1/LTBP3	T	-0·0360	0·0050	110000	49	Köttgen[s8]
21	rs653178	12	110492139	ATXN2/PTPN11	T	-0·0350	0·0050	110000	49	Köttgen[s8]
22	rs1394125	15	73946038	UBE2Q2/NRG4	A	0·0430	0·0060	110000	49	Köttgen[s8]
23	rs6598541	15	97088658	IGF1R/IGF1R	A	0·0430	0·0060	110000	49	Köttgen[s8]
24	rs7193778	16	68121391	NFAT5/NFAT5	T	-0·0460	0·0080	110000	49	Köttgen[s8]
25	rs7188445	16	78292488	MAF/MAF	A	-0·0320	0·0050	110000	49	Köttgen[s8]
26	rs7224610	17	50719787	HLF/HLF	A	-0·0420	0·0050	110000	49	Köttgen[s8]
27	rs742132	6	25715550	LRRC16A/LRRC16A	A	0·0540	0·0092	27923	14	Kolz[s9]
28	rs2307394	2	148432898	ORC4L/ACVR2A	T	-0·0290	0·0050	110000	49	Köttgen[s8]
29	rs17786744	8	23832951	STC1/STC1	A	-0·0290	0·0050	110000	49	Köttgen[s8]
30	rs2079742	17	56820479	BCAS3/C17orf82	T	0·0430	0·0080	110000	49	Köttgen[s8]
31	rs164009	17	71795264	QRICH2/PRPSAP1	A	0·028	0·005	110000	49	Köttgen[s8]

[a] Units are SD uric acid per copy of effect allele using a population SD for uric acid of 90·7 µmol/L (=1·5 mg/dl) reported by CHARGE. (Yang et al.).[2]

Table S4. Association of the 31 SNP plasma urate instrument with cardiovascular traits.

Cardio-vascular trait*	Difference in risk factor per inverse variance weighted allele.	95%CI
HDL-C (mmol/L)	-0.0079	-0.0096, -0.0062
LDL-C (mmol/L)	-0.0014	-0.0032, 0.0005
TC (mmol/L)	0.0003	-0.0015, 0.0021
TG (mmol/L)	0.0142	0.0125, 0.0158
SBP (mm Hg)	0.0045	0.0026, 0.0064
DBP (mm Hg)	0.0054	0.0033, 0.0074
Fasting glucose (mmol/L)	-0.0010	-0.0026, 0.0006
BMI (kg/m^2)	-0.0003	-0.0008, 0.0002
Diabetes (OR)	0.9991	0.992, 1.0064

* See Table S2 for numbers of individuals and studies

Table S5. Proxy SNPs used in the Instrument.

**Index**	**Lead SNP**	**Proxy SNP**	**R^2**
**3**	rs12498742	rs734553	0.89
**6**	rs1165151	rs1183201	1
**9**	rs478607	rs505802	0.44

Table S6. Power (two-sided α=0·05) for conventional IV regression of the binary outcomes.

Outcome	Proportion cases	Observational OR (per SD urate)	R^2 of instrument	N required for 80% power	Actual n	Power at actual n
CHD	0·317	1·07	0·042	183868	198598	0.83
T2D	0·175	1·32	0·042	13910	84638	1

Table S7. Power (α=0·05) for conventional IV regression of the continuous outcomes.

Outcome (units)	β_{yx}(true)[a]	R^2	N required for 80% power	Actual n	Power at actual n
LDL-C (mmol/L)	0·073	0·042	34882	196621	1
HDL-C (mmol/L)	-0·183	0·042	5394	196621	1
TC (mmol/L)	0·129	0·042	11044	196621	1
TG (mmol/L)	0·265	0·042	2475	196621	1
SBP (mmHg)	0.163	0·042	6847	89667	1
DBP (mmHg)	0.169	0·042	6357	89667	1
Fasting Glucose (mmol/L)	-0·039	0·042	122697	57397	0·48

Table S8. Gene Ontology Enrichment Analysis.

Term (GO reference)	Background frequency	Sample frequency	Bonferroni corrected P-value
urate metabolic process (GO:0046415)	13	7	3.96E-13
purine-containing compound metabolic process (GO:0072521)	311	8	5.57E-05
heterocycle metabolic process (GO:0046483)	4328	19	1.84E-03
cellular aromatic compound metabolic process (GO:0006725)	4332	19	1.87E-03
organic cyclic compound metabolic process (GO:1901360)	4571	19	4.51E-03
cellular nitrogen compound metabolic process (GO:0034641)	4598	19	4.97E-03
nitrogen compound metabolic process (GO:0006807)	5014	19	2.01E-02

[a] See Table S2 for sources of data used to estimate the regression coefficient. Units are SD/SD.

Table S9. Function and druggability of genes represented in the genetic instrument for plasma urate.

SNP	CHR	GENE (nearest/GRAIL)	Drugs	Gene function (from; http://www.genecards.org/)
rs1471633	1	PDZK1/PDZK1	None	PDZK1: This gene encodes a PDZ domain-containing scaffolding protein. PDZ domain-containing molecules bind to and mediate the subcellular localization of target proteins. The encoded protein mediates the localization of cell surface proteins and plays a critical role in cholesterol metabolism by regulating the HDL receptor, scavenger receptor class B type 1. Single nucleotide polymorphisms in this gene may be associated with metabolic syndrome, and overexpression of this gene may play a role in drug resistance of multiple myeloma. Pseudogenes of this gene are located on the long arm of chromosome 1. Alternatively spliced transcript variants encoding multiple isoforms have been observed for this gene.
rs1260326	2	GCKR/GCKR	In development. 61	This gene encodes a protein belonging to the GCKR subfamily of the SIS (Sugar ISomerase) family of proteins. The gene product is a regulatory protein that inhibits glucokinase in liver and pancreatic islet cells by binding non-covalently to form an inactive complex with the enzyme. This gene is considered a susceptibility gene candidate for a form of maturity-onset diabetes of the young (MODY).
rs12498742	4	SLC2A9/SLC2A9	None	This gene encodes a member of the SLC2A facilitative glucose transporter family. Members of this family play a significant role in maintaining glucose homeostasis. The encoded protein may play a role in the development and survival of chondrocytes in cartilage matrices. Two transcript variants encoding distinct isoforms have been identified for this gene.
rs2231142	4	ABCG2/ABCG2		The membrane-associated protein encoded by this gene is included in the superfamily of ATP-binding cassette (ABC) transporters. ABC proteins transport various molecules across extra- and intra-cellular membranes. ABC genes are divided into seven distinct subfamilies (ABC1, MDR/TAP, MRP, ALD, OABP, GCN20, White). This protein is a member of the White subfamily. Alternatively referred to as a breast cancer resistance protein, this protein functions as a xenobiotic transporter which may play a major role in multi-drug resistance. It likely serves as a cellular defense mechanism in response to mitoxantrone and anthracycline exposure. Significant expression of this protein has been observed in the placenta, which may suggest a potential role for this molecule in placenta tissue. Multiple transcript variants encoding different isoforms have been found for this gene.
rs675209	6	RREB1/RREB1	None	RREB1: The protein encoded by this gene is a zinc finger transcription factor that binds to RAS-responsive elements (RREs) of gene promoters. It has been shown that the calcitonin gene promoter contains an RRE and that the encoded protein binds there and increases expression of calcitonin, which may be involved in Ras/Raf-mediated cell differentiation. Multiple transcript variants encoding several different isoforms have been found for this gene. LY86: May cooperate with CD180 and TLR4 to mediate the innate immune response to bacterial lipopolysaccharide (LPS) and cytokine production. Important for efficient CD180 cell surface expression (By similarity)
rs1165151	6	SLC17A1/SLC17A3	None	SLC17A1 (solute carrier family 17 (organic anion transporter), member 1) is a protein-coding gene. Diseases associated with SLC17A1 include cardiovascular disease risk factor. GO annotations related to this gene include sodium-dependent phosphate transmembrane transporter activity and symporter activity. An important paralog of this gene is SLC17A7.
rs1171614	10	SLC16A9/SLC16A9	None	SLC16A9 (solute carrier family 16, member 9) is a protein-coding gene. GO annotations related to this gene include symporter activity. An important paralog of this gene is SLC16A4.

SNP	CHR	GENE (nearest/GRAIL)	Drugs	Gene function (from; http://www.genecards.org/)
rs2078267	11	SLC22A11/ SLC22A11	Probenecid	SLC22A11 (solute carrier family 22 (organic anion/urate transporter), member 11) is a protein-coding gene. Diseases associated with SLC22A11 include cardiovascular disease risk factor. GO annotations related to this gene include inorganic anion exchanger activity and sodium-independent organic anion transmembrane transporter activity. An important paralog of this gene is SLC22A5.
rs478607	11	NRXN2/SLC22A12	Sulfinpyrazone	SLC22A12 (solute carrier family 22 (organic anion/urate transporter), member 12) is a protein-coding gene. Diseases associated with SLC22A12 include renal hypouricemia 1, and renal hypouricemia. GO annotations related to this gene include PDZ domain binding and urate transmembrane transporter activity. An important paralog of this gene is SLC22A11.
rs3741414	12	INHBC/INHBE	None	INHBC (inhibin, beta C) is a protein-coding gene. Diseases associated with INHBC include gastric diffuse adenocarcinoma, and endometrial adenocarcinoma. GO annotations related to this gene include growth factor activity and transforming growth factor beta receptor binding. An important paralog of this gene is GDF11. / INHBE: (inhibin, beta E) is a protein-coding gene. Diseases associated with INHBE include endometrial adenocarcinoma, and germ cell tumors. GO annotations related to this gene include growth factor activity and hormone activity. An important paralog of this gene is GDF11.
rs11264341	1	TRIM46/PKLR	None/compounds in development	TRIM46: Protein coding. Paaralog is TRIM13 which is associated with leukemia. PKLR: The protein encoded by this gene is a pyruvate kinase that catalyzes the transphosphorylation of phohsphoenolpyruvate into pyruvate and ATP, which is the rate-limiting step of glycolysis. Associated with hemolytic anemia.
rs17050272	2	INHBB/INHBB	None	INHBB: A protein-coding gene. Diseases associated with INHBB include varicocele, and ectopic pregnancy. GO annotations related to this gene include growth factor activity and protein homodimerization activity. An important paralog of this gene is GDF11.
rs6770152	3	SFMBT1/MUSTN1	None/None	SFMBT1: (Scm-like with four mbt domains 1) is a protein-coding gene. Diseases associated with SFMBT1 include normal pressure hydrocephalus, and acute poststreptococcal glomerulonephritis. GO annotations related to this gene include histone binding and transcription corepressor activity. An important paralog of this gene is L3MBTL1./MUSTN1: May be involved in the development and regeneration of the musculoskeletal system (By similarity)
rs17632159	5	TMEM171/TMEM171	None	Transmembrane protein.
rs729761	6	VEGFA/VEGFA	Pegaptanib Sodium(Top), Ranibizumab(Top), Aflibercept(Top), Bevacizumab(Can)	Growth factor active in angiogenesis, vasculogenesis and endothelial cell growth. Induces endothelial cell proliferation, promotes cell migration, inhibits apoptosis and induces permeabilization of blood vessels. Binds to the FLT1/VEGFR1 and KDR/VEGFR2 receptors, heparan sulfate and heparin. NRP1/Neuropilin-1 binds isoforms VEGF-165 and VEGF-145. Isoform VEGF165B binds to KDR but does not activate downstream signaling pathways, does not activate angiogenesis and inhibits tumor growth.
rs1178977	7	BAZ1B/MLXIPL	None/None	BAZ1B: (bromodomain adjacent to zinc finger domain, 1B) is a protein-coding gene. Diseases associated with BAZ1B include williams-beuren syndrome, and williams syndrome. GO annotations related to this gene include chromatin binding and non-membrane spanning protein tyrosine kinase activity. An important paralog of this gene is BAZ1A./MLXIPL: This gene encodes a basic helix-loop-helix leucine zipper transcription factor of the Myc/Max/Mad superfamily. This protein forms a heterodimeric complex and binds and activates, in a glucose-dependent manner, carbohydrate response element

SNP	CHR	GENE (nearest/GRAIL)	Drugs	Gene function (from; http://www.genecards.org/)
				(ChoRE) motifs in the promoters of triglyceride synthesis genes. The gene is deleted in Williams-Beuren syndrome, a multisystem developmental disorder caused by the deletion of contiguous genes at chromosome 7q11.23.
rs10480300	7	PRKAG2/PRKAG2	None	PRKAG2 (protein kinase, AMP-activated, gamma 2 non-catalytic subunit) is a protein-coding gene. Diseases associated with PRKAG2 include cardiomyopathy, familial hypertrophic 6, and wolff-parkinson-white syndrome. GO annotations related to this gene include protein kinase binding and cAMP-dependent protein kinase regulator activity. An important paralog of this gene is PRKAG1
rs2941484	8	HNF4G/HNF4G	None	HNF4G (hepatocyte nuclear factor 4, gamma) is a protein-coding gene. GO annotations related to this gene include steroid hormone receptor activity and sequence-specific DNA binding transcription factor activity. An important paralog of this gene is RXRA.
rs10821905	10	A1CF/ASAH2	None	A1CF: Essential component of the apolipoprotein B mRNA editing enzyme complex which is responsible for the postranscriptional editing of a CAA codon for Gln to a UAA codon for stop in APOB mRNA. Binds to APOB mRNA and is probably responsible for docking the catalytic subunit, APOBEC1, to the mRNA to allow it to deaminate its target cytosine. The complex also protects the edited APOB mRNA from nonsense-mediated decay/ASAH2: Hydrolyzes the sphingolipid ceramide into sphingosine and free fatty acid at an optimal pH of 6.5-8.5. Acts as a key regulator of sphingolipid signaling metabolites by generating sphingosine at the cell surface. Acts as a repressor of apoptosis both by reducing C16-ceramide, thereby preventing ceramide-induced apoptosis, and generating sphingosine, a precursor of the antiapoptotic factor sphingosine 1-phosphate. Probably involved in the digestion of dietary sphingolipids in intestine by acting as a key enzyme for the catabolism of dietary sphingolipids and regulating the levels of bioactive sphingolipid metabolites in the intestinal tract.
rs642803	11	OVOL1/LTBP3	None	OVOL1: Putative transcription factor. Involved in hair formation and spermatogenesis. May function in the differentiation and/or maintenance of the urogenital system (By similarity)/LTBP3: May be involved in the assembly, secretion and targeting of TGFB1 to sites at which it is stored and/or activated. May play critical roles in controlling and directing the activity of TGFB1. May have a structural role in the extra cellular matrix (ECM)
rs653178	12	ATXN2/PTPN11	None/Enoxolone	ATXN2: Involved in EGFR trafficking, acting as negative regulator of endocytic EGFR internalization at the plasma membrane./PTPN11: Acts downstream of various receptor and cytoplasmic protein tyrosine kinases to participate in the signal transduction from the cell surface to the nucleus. Dephosphorylates ROCK2 at Tyr-722 resulting in stimulatation of its RhoA binding activity.
rs1394125	15	UBE2Q2/NRG4	None	UBE2Q2: Accepts ubiquitin from the E1 complex and catalyzes its covalent attachment to other proteins. In vitro catalyzes 'Lys-48'-linked polyubiquitination/ NRG4: Low affinity ligand for the ERBB4 tyrosine kinase receptor. Concomitantly recruits ERBB1 and ERBB2 coreceptors, resulting in ligand-stimulated tyrosine phosphorylation and activation of the ERBB receptors. Does not bind to the ERBB1, ERBB2 and ERBB3 receptors (By similarity)
rs6598541	15	IGF1R/IGF1R	Mecasermin Rinfabate (Igf), Mecasermin(Igf)	IGF1R: IGF1R (insulin-like growth factor 1 receptor) is a protein-coding gene. Diseases associated with IGF1R include insulin-like growth factor 1 resistance to, and insulin-like growth factor i deficiency. GO annotations related to this gene include insulin receptor binding and identical protein binding. An important paralog of this gene is ROR1.
rs7193778	16	NFAT5/NFAT5	None	Transcription factor involved in the transcriptional regulation of osmoprotective and inflammatory genes. Regulates hypertonicity-

SNP	CHR	GENE (nearest/GRAIL)	Drugs	Gene function (from; http://www.genecards.org/)
				induced cellular accumulation of osmolytes
rs7188445	16	MAF/MAF	None	MAF (v-maf avian musculoaponeurotic fibrosarcoma oncogene homolog) is a protein-coding gene. Diseases associated with MAF include nephrogenic adenofibroma, and plasma cell leukemia. GO annotations related to this gene include sequence-specific DNA binding and sequence-specific DNA binding transcription factor activity. An important paralog of this gene is NRL.
rs7224610	17	HLF/HLF	None	HLF (hepatic leukemia factor) is a protein-coding gene. Diseases associated with HLF include leukemia, acute lymphoblastic 3, and acute lymphoblastic leukemia. GO annotations related to this gene include double-stranded DNA binding and sequence-specific DNA binding transcription factor activity. An important paralog of this gene is DBP.
rs742132	6	LRRC16A/LRRC16A	None	LRRC16A (leucine rich repeat containing 16A) is a protein-coding gene. Diseases associated with LRRC16A include acute urate nephropathy. An important paralog of this gene is LRRC16B.
rs2307394	2	ORC4L/ACVR2A	None/Dasatinib,Lestaurtinib,Alvocidib	ORC4 (origin recognition complex, subunit 4) is a protein-coding gene. Diseases associated with ORC4 include meier-gorlin syndrome 2, and meier-gorlin syndrome. GO annotations related to this gene include DNA replication origin binding and nucleotide binding./ACVR2A (activin A receptor, type IIA) is a protein-coding gene. Diseases associated with ACVR2A include multiple synostoses syndrome. GO annotations related to this gene include PDZ domain binding and growth factor binding. An important paralog of this gene is ACVR1C.
rs17786744	8	STC1/STC1	None	STC1 (stanniocalcin 1) is a protein-coding gene. Diseases associated with STC1 include pheochromocytoma, and fibrosarcoma. GO annotations related to this gene include hormone activity. An important paralog of this gene is STC2. The protein may play a role in the regulation of renal and intestinal calcium and phosphate transport, cell metabolism, or cellular calcium/phosphate homeostasis.
rs2079742	17	BCAS3/C17orf82	None	BCAS3 (breast carcinoma amplified sequence 3) is a protein-coding gene. Diseases associated with BCAS3 include breast cancer./C17orf82 (chromosome 17 open reading frame 82) is a protein-coding gene.
rs164009	17	QRICH2/PRPSAP1	None	QRICH2 (glutamine rich 2) is a protein-coding gene./ PRPSAP1 (phosphoribosyl pyrophosphate synthetase-associated protein 1) is a protein-coding gene. GO annotations related to this gene include enzyme inhibitor activity and magnesium ion binding. An important paralog of this gene is PRPS1.

Table S10. Sensitivity tests with different covariate models.

Outcome/Exposure	Covariates	Point estimate: IV (OR) (95% CI) from MVMR with specified model; full data.	Mean (median) estimate from sensitivity test in which the model was fitted 100,000 times removing 6 SNPs at random from the data in each cycle.	95% range of estimates from sensitivity test.	% of estimates from the sensitivity test which lie outside the confidence interval of the IV regression.
CHD/Urate	-	1.177 (1.076, 1.286)	1.184 (1.176)	1.122, 1.299	4.85
CHD/Urate	HDL	1.094 (0.991, 1.208)	1.096 (1.094)	1.044, 1.168	0.34
CHD/Urate	TG	1.173 (1.068, 1.289)	1.18 (1.169)	1.111, 1.314	6.43*
CHD/Urate	DBP	1.097 (1, 1.202)	1.098 (1.103)	1.022, 1.166	0.92
CHD/Urate	SBP	1.121 (1.024, 1.227)	1.118 (1.128)	1.008, 1.176	2.89
CHD/Urate	SBP+HDL	1.111 (1.006, 1.227)	1.108 (1.119)	0.996, 1.171	2.77
CHD/Urate	SBP+TG	1.136 (1.033, 1.249)	1.134 (1.141)	1.031, 1.216	2.83
CHD/Urate	SBP+DBP	1.101 (1.003, 1.208)	1.101 (1.108)	1.013, 1.166	2.12
CHD/Urate	HDL+TG	1.102 (0.999, 1.217)	1.103 (1.099)	1.045, 1.195	0.75
CHD/Urate	HDL+DBP	1.09 (0.987, 1.203)	1.091 (1.095)	1.018, 1.165	1.04
CHD/Urate	TG+DBP	1.107 (1.005, 1.218)	1.107 (1.111)	1.028, 1.2	1.53
CHD/Urate	TG+DBP+HDL	1.094 (0.991, 1.208)	1.092 (1.095)	1.016, 1.18	1.34
CHD/Urate	SBP+HDL+DBP	1.095 (0.991, 1.211)	1.095 (1.101)	1.006, 1.169	2.04
CHD/Urate	SBP+TG+DBP	1.114 (1.011, 1.228)	1.112 (1.118)	1.023, 1.202	2.13
CHD/Urate	SBP+HDL+TG	1.116 (1.011, 1.232)	1.111 (1.118)	1.017, 1.19	2.42
CHD/Urate	SBP+HDL+TG+DBP	1.101 (0.996, 1.218)	1.096 (1.101)	1.013, 1.185	1.89
CHD/Urate	MR Egger method	1.049 (0.918, 1.200)	1.035 (1.045)	0.699, 1.134	3.81

* Distribution of sensitivity test does not fit within the assumed normal distribution of the point estimate in full data for the model. This indicates that the model is sensitive to SNP selection and the confidence interval on the point estimate is anti-conservative. Conversely if the value is less than 5% it suggests the model is insensitive to SNP selection and the interval on the point estimate is likely to be conservative.

Figure S1. The distribution of plasma urate concentration in the UCLEB consortium data.

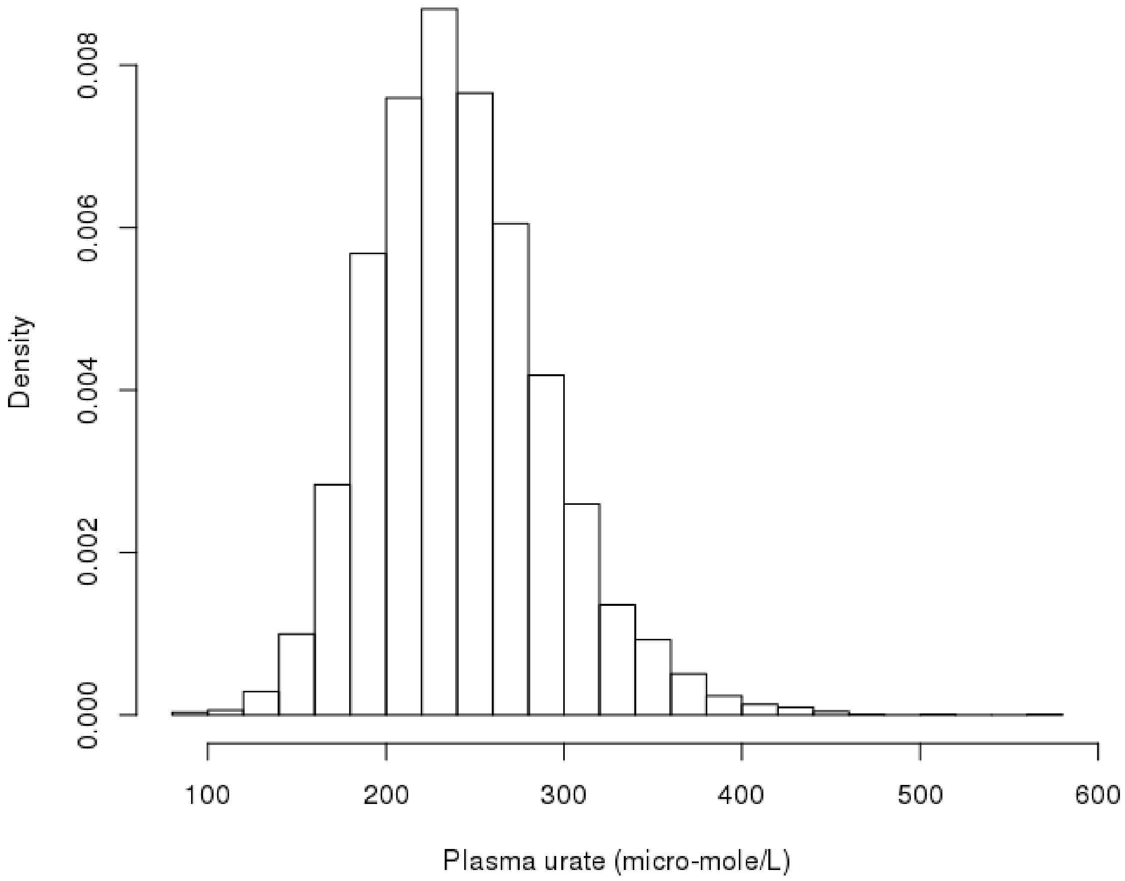

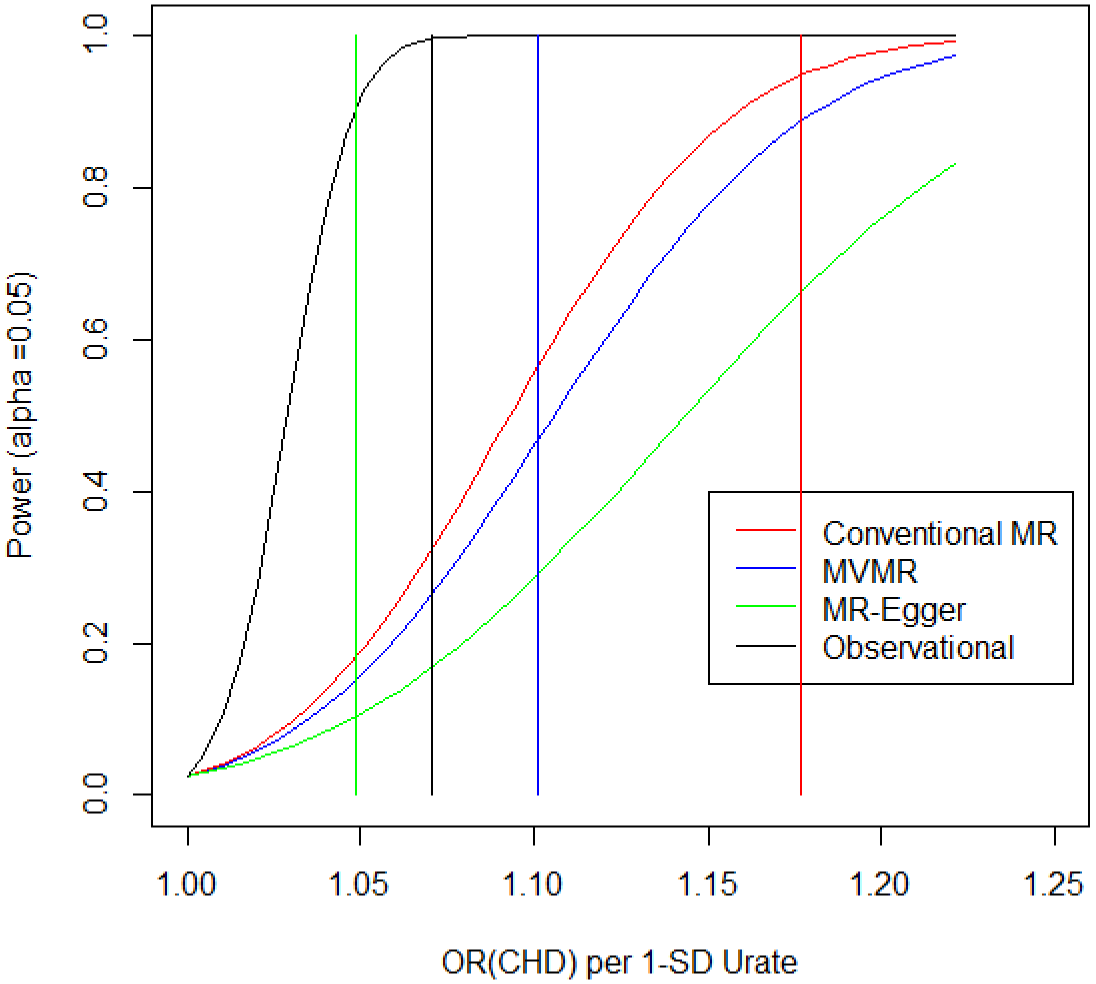

Figure S2. Power curves derived from analytical outcomes. The vertical lines represent the effect, estimated by each method, of plasma urate concentration on CHD risk, colour coded as legend.

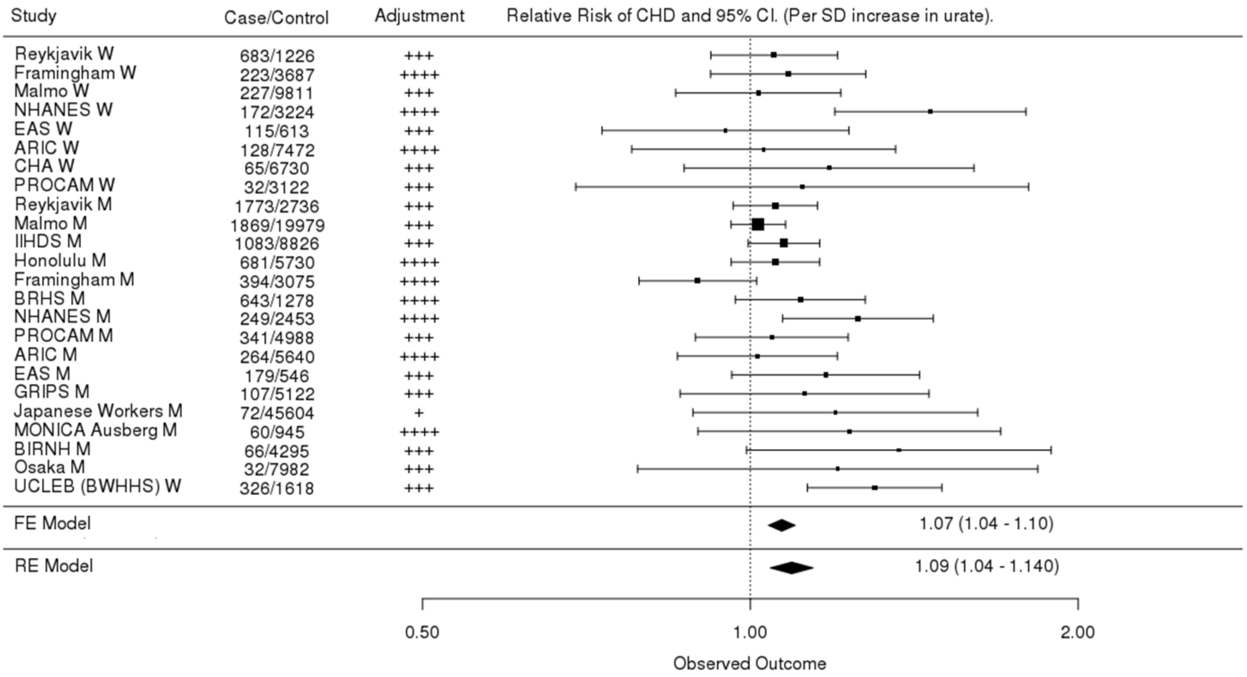

Figure S3. Observational association between plasma urate concentration and relative risk of CHD in 17 prospective population-based cohorts. Summary estimates obtained by fixed-effects (FE) and random effects (RE) meta-analysis are presented. Adjustment: + age and sex; +++ age, sex, smoking and some additional risk factors (not specified by original author), ++++ as +++ with adjustment for pre-existing CHD. Apart from UCLEB (BWHHS) the data were obtained from Wheeler et al. 2005,[s18] the order of studies mirrors that publication.. (Size of point markers is proportional to the inverse variance). W = Women, M = Men.

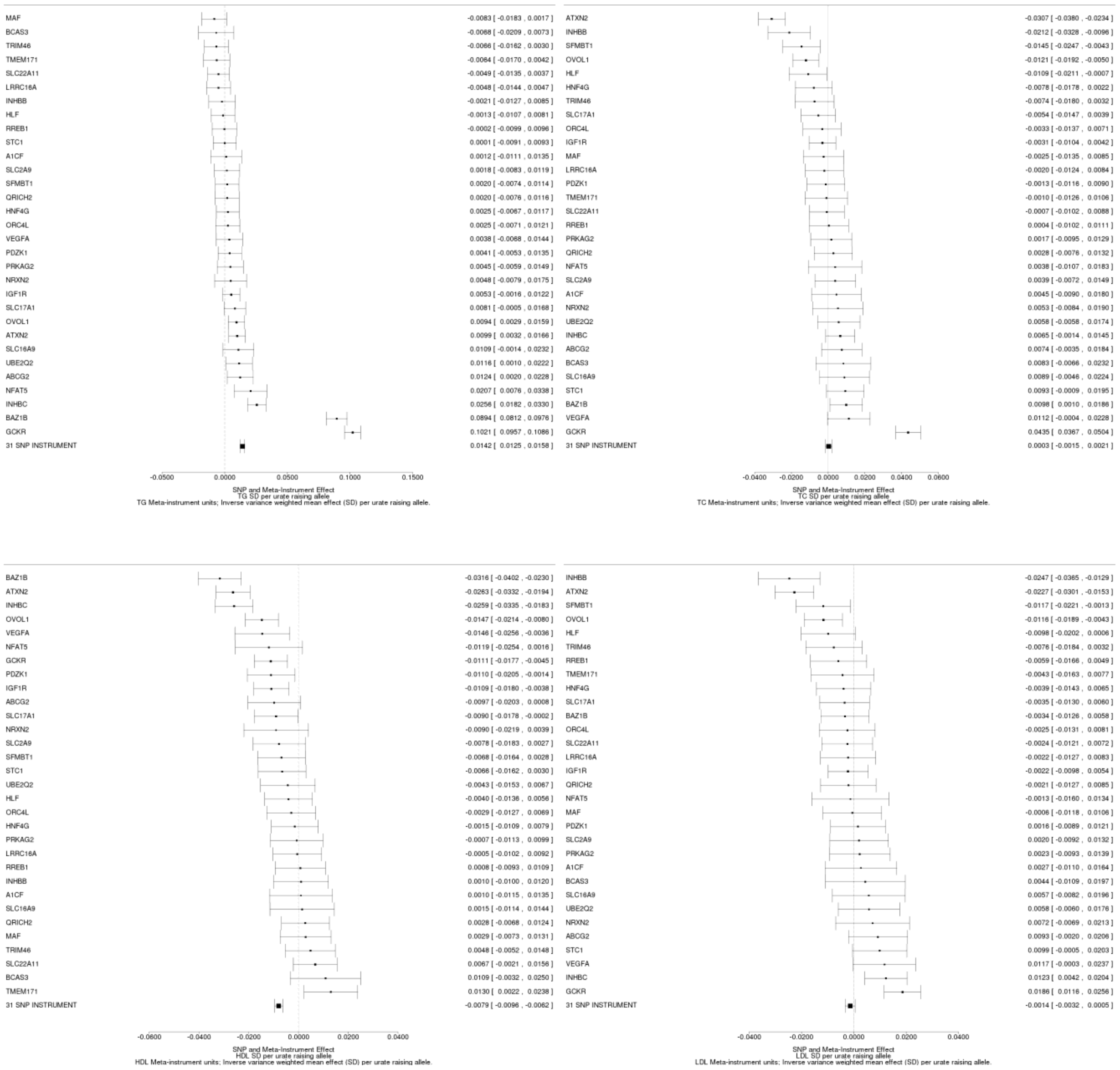

Figure S4. The association of individual SNPs and the 31 SNP instrument for plasma urate concentration with continuous phenotypes. (Error bars are 95%CI, SNP order is by magnitude of effect within a phenotype, and all effects are with respect to the urate raising allele). Significant association in the 31 SNP instrument is indicative of pleiotropy.

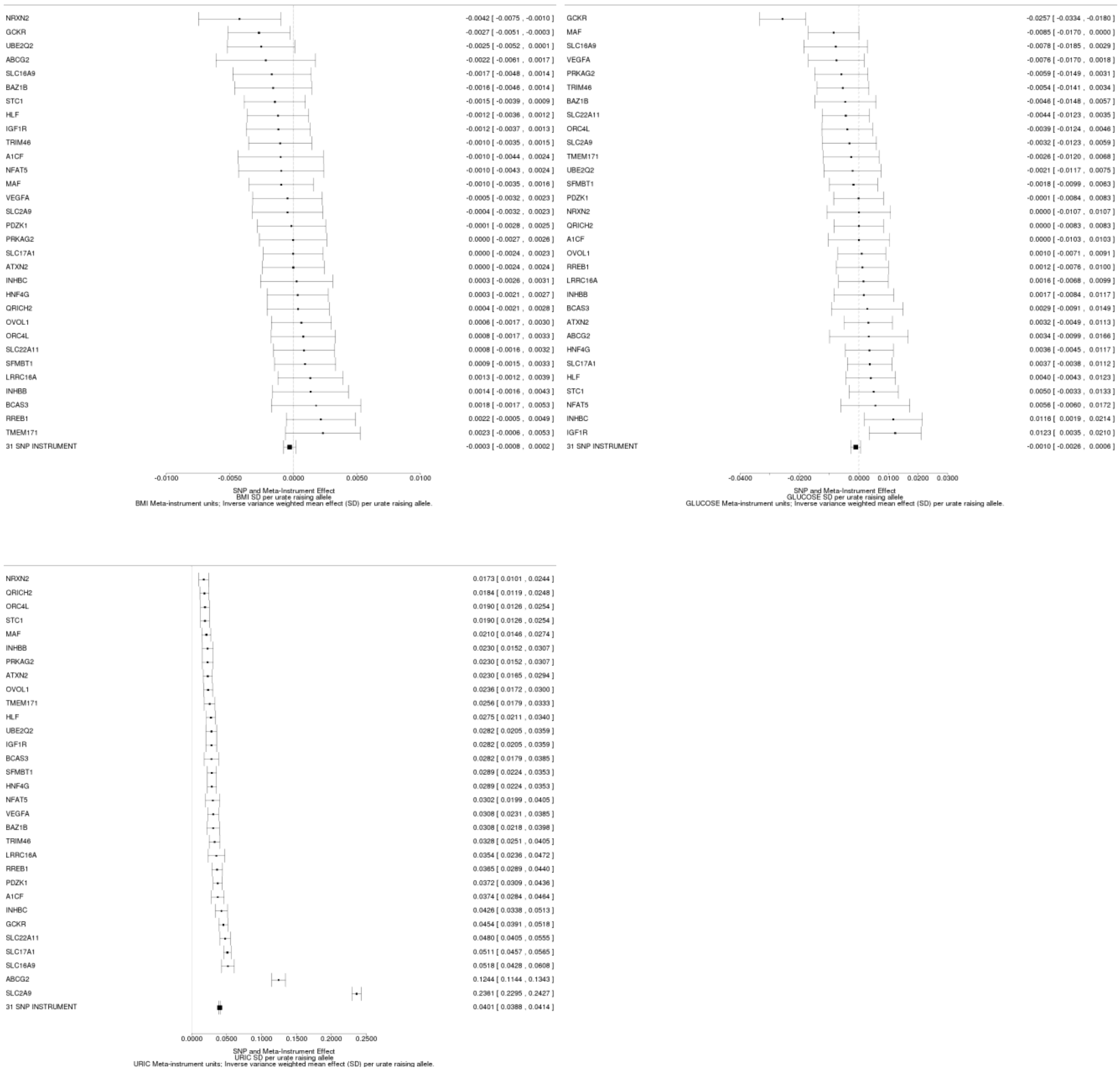

Figure S5. The association of individual SNPs and the 31 SNP instrument for plasma urate concentration with continuous phenotypes. (Error bars are 95%CI, SNP order is by magnitude of effect within a phenotype, and all effects are with respect to the urate raising allele). Significant association in the 31 SNP instrument is indicative of pleiotropy.

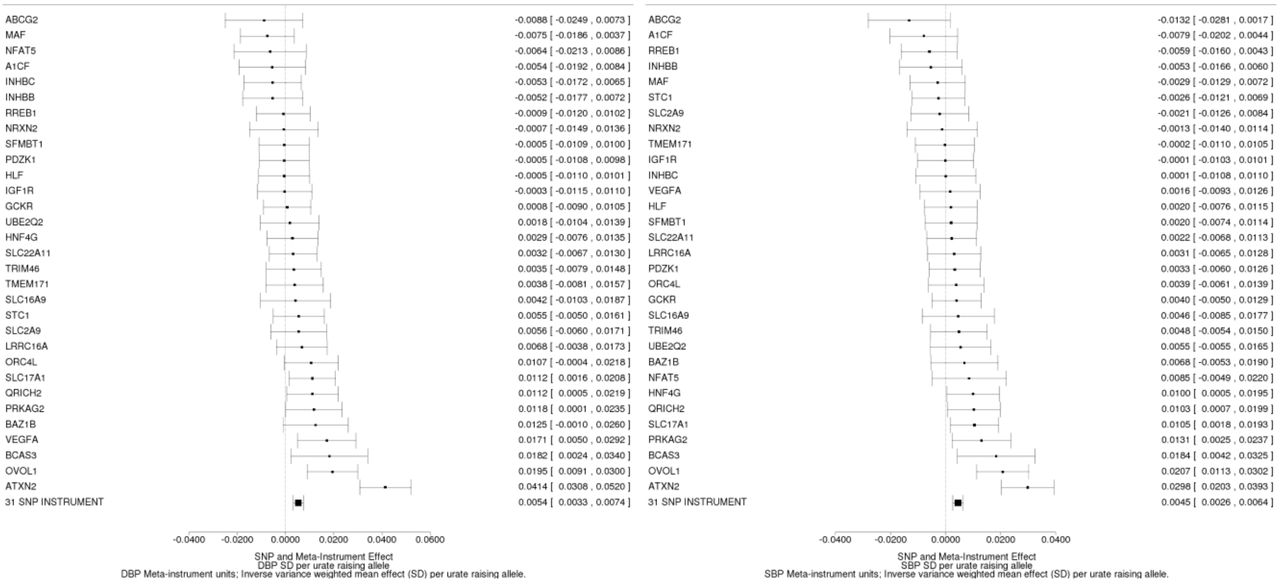

Figure S6. The association of the individual SNPs and the 31 SNP instrument for plasma urate concentration with blood pressure. (Error bars are 95%CI, SNP order is by magnitude of effect within a phenotype, and all effects are with respect to the urate raising allele). Significant association in the 31 SNP instrument is indicative of pleiotropy.

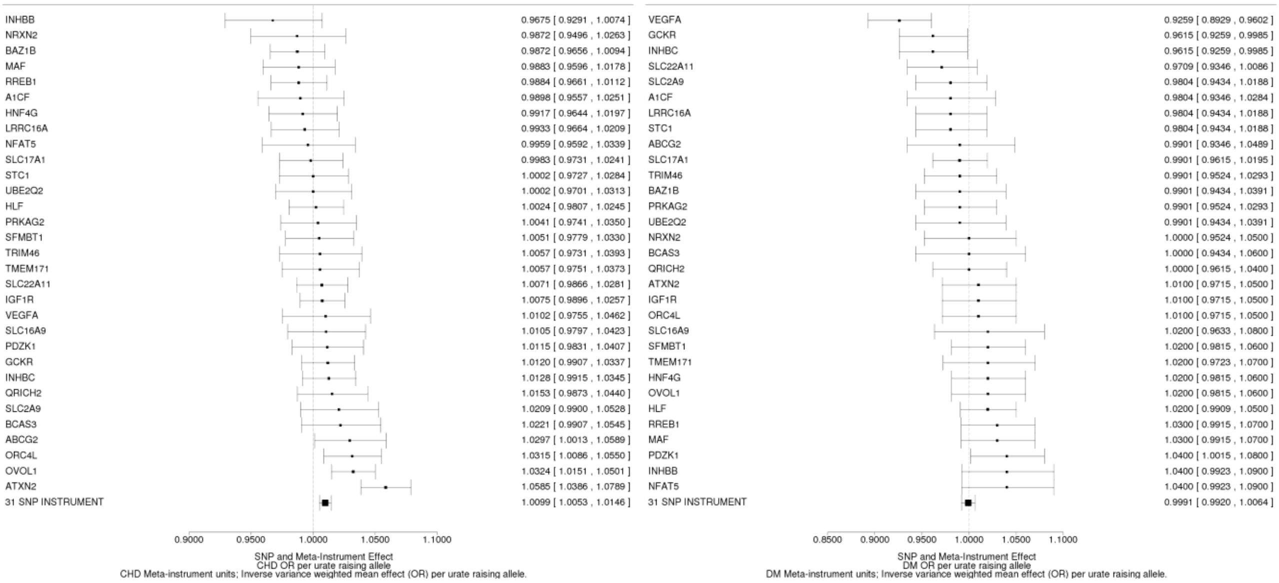

Figure S7. The association of the individual SNPs and the 31 SNP instrument for plasma urate concentration with binary phenotypes. (Error bars are 95%CI, SNP order is by magnitude of effect within a phenotype, and all effects are with respect to the urate raising allele). Significant association in the 31 SNP instrument is indicative of pleiotropy.

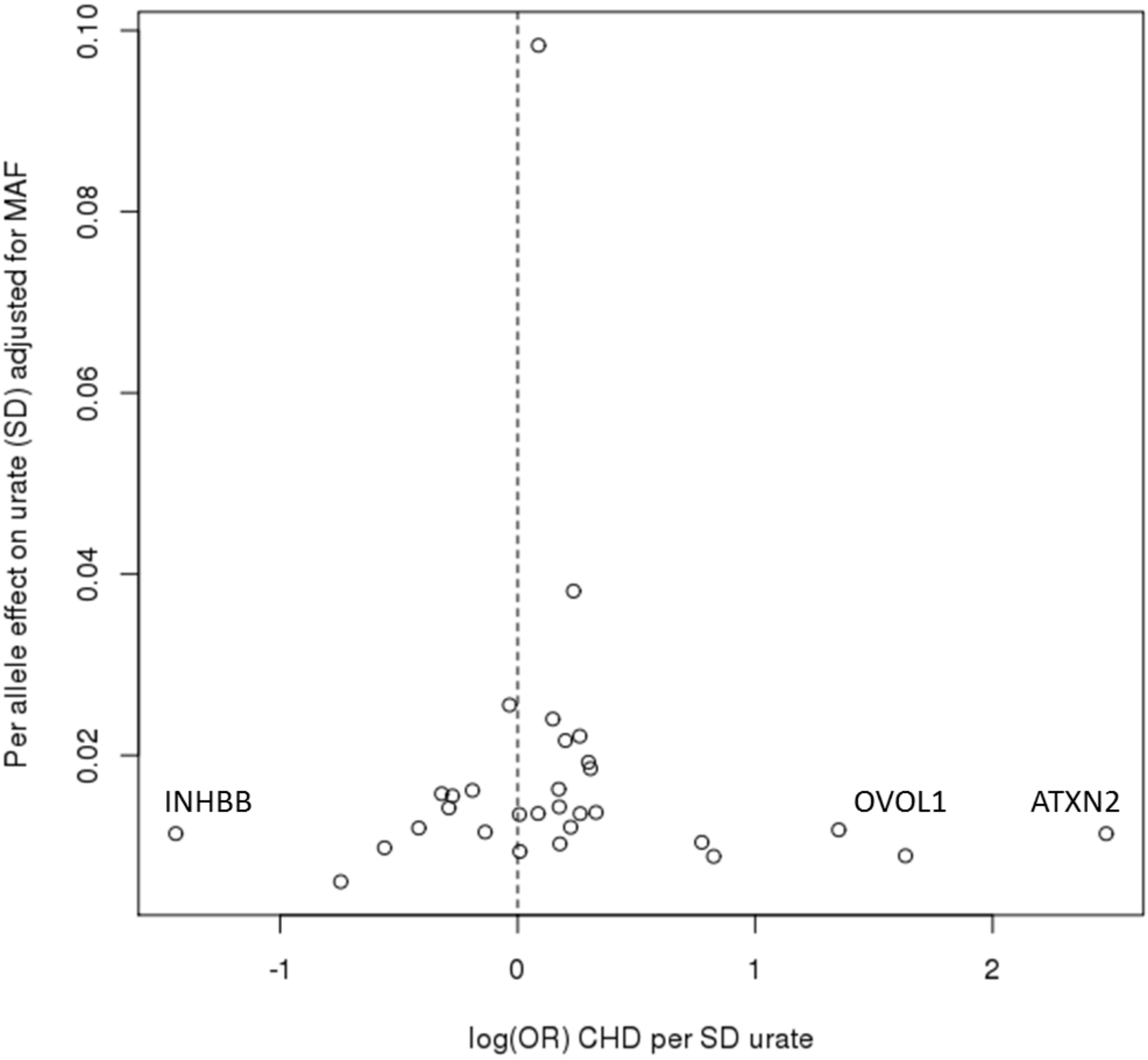

Figure S8. Funnel plot of individual IV beta estimates for SNPs in the instrument. The distribution about the point estimate is asymmetric suggesting there is an unmeasured net pleiotropic effect on the instrument. (Egger test for funnel plot symmetry P.value = 0.011).

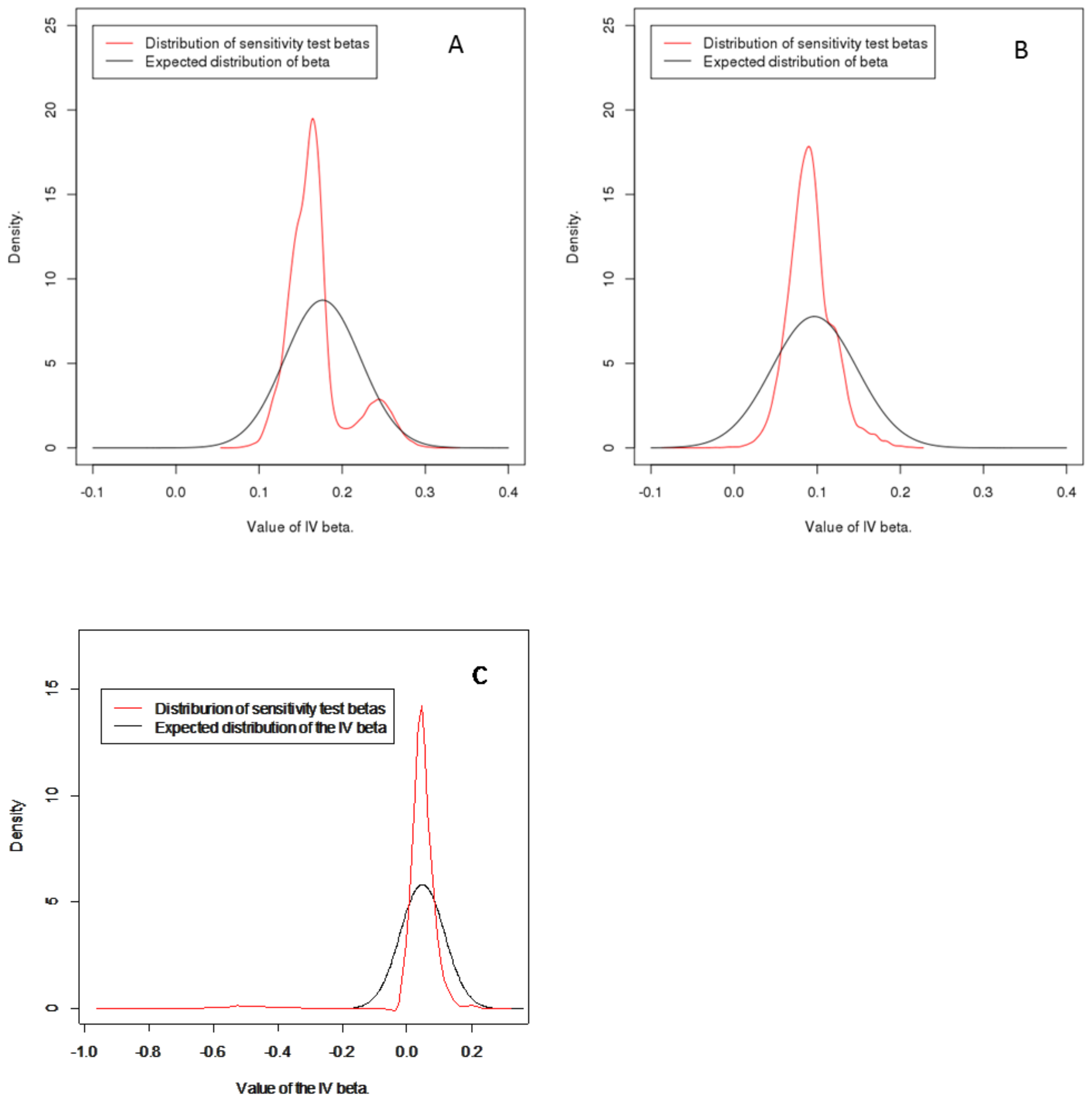

Figure S9. Sensitivity tests. The assumed normal distribution (black) of the point estimate of the IV beta using the 31 SNP instrument with (B) and without (A) covariates. Similar for MR Egger regression(C). In each case the red curve is the empirical distribution of the IV beta estimated in 100 000 25 SNP instruments obtained by repeatedly excluding 6 SNPs at random.

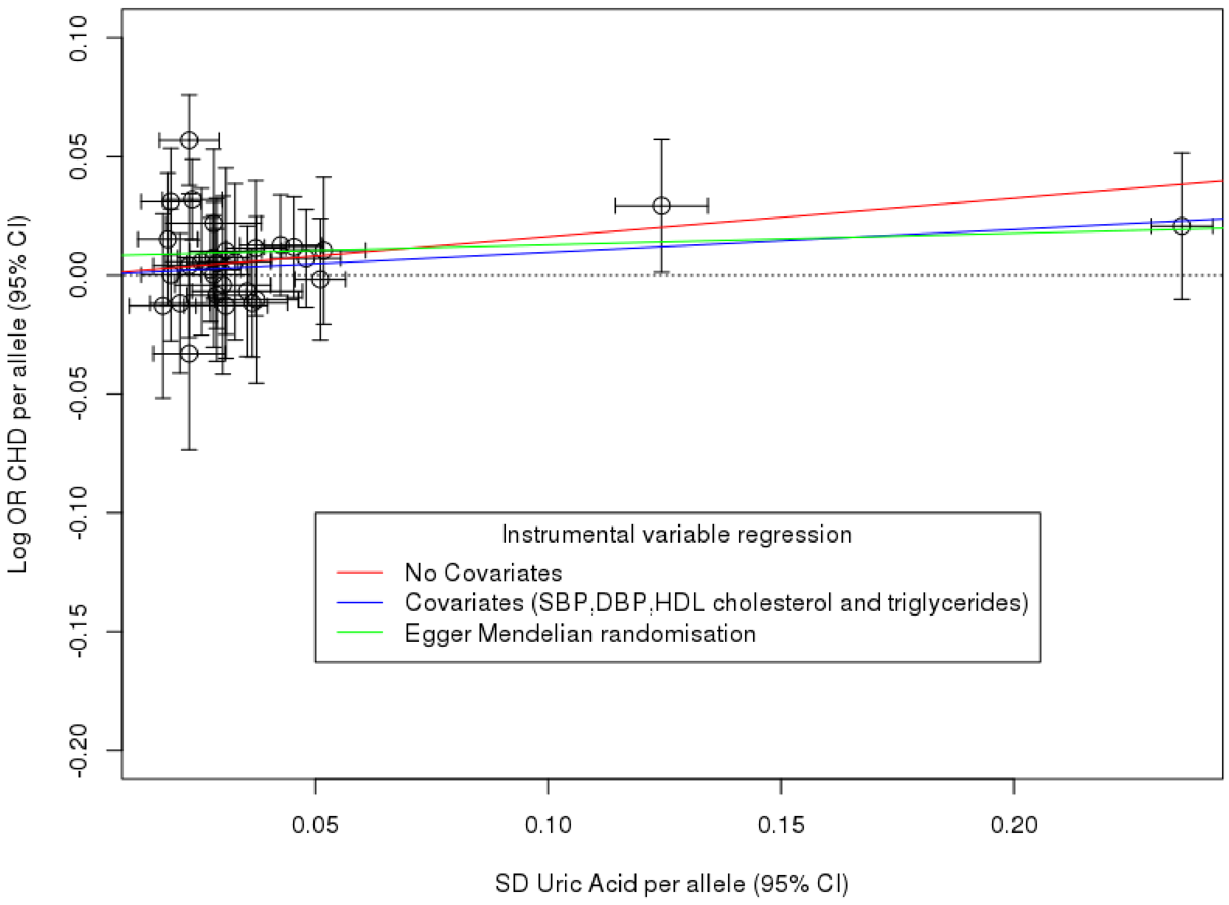

Figure S10. Association of SNPs with plasma urate concentration and risk of coronary heart disease. Effect estimates are as figure 2 in the main paper except that in this figure all effect estimates have been adjusted to be relative to the urate raising allele of the SNP. This allows the Egger Mendelian randomisation to be represented in the same figure. Error bars are 95% CI in each dimension.

Supplementary Appendix 1. Contributors to the ICBP[s20]

Ehret GB, Munroe PB, Rice KM, Bochud M, Johnson AD, Chasman DI, Smith AV, Tobin MD, Verwoert GC, Hwang SJ, Pihur V, Vollenweider P, O'Reilly PF, Amin N, Bragg-Gresham JL, Teumer A, Glazer NL, Launer L, Zhao JH, Aulchenko Y, Heath S, Sõber S, Parsa A, Luan J, Arora P, Dehghan A, Zhang F, Lucas G, Hicks AA, Jackson AU, Peden JF, Tanaka T, Wild SH, Rudan I, Igl W, Milaneschi Y, Parker AN, Fava C, Chambers JC, Fox ER, Kumari M, Go MJ, van der Harst P, Kao WH, Sjögren M, Vinay DG, Alexander M, Tabara Y, Shaw-Hawkins S, Whincup PH, Liu Y, Shi G, Kuusisto J, Tayo B, Seielstad M, Sim X, Nguyen KD, Lehtimäki T, Matullo G, Wu Y, Gaunt TR, Onland-Moret NC, Cooper MN, Platou CG, Org E, Hardy R, Dahgam S, Palmen J, Vitart V, Braund PS, Kuznetsova T, Uiterwaal CS, Adeyemo A, Palmas W, Campbell H, Ludwig B, Tomaszewski M, Tzoulaki I, Palmer ND, CARDIoGRAM consortium, CKDGen Consortium, KidneyGen Consortium, EchoGen consortium, CHARGE-HF consortium, Aspelund T, Garcia M, Chang YP, O'Connell JR, Steinle NI, Grobbee DE, Arking DE, Kardia SL, Morrison AC, Hernandez D, Najjar S, McArdle WL, Hadley D, Brown MJ, Connell JM, Hingorani AD, Day IN, Lawlor DA, Beilby JP, Lawrence RW, Clarke R, Hopewell JC, Ongen H, Dreisbach AW, Li Y, Young JH, Bis JC, Kähönen M, Viikari J, Adair LS, Lee NR, Chen MH, Olden M, Pattaro C, Bolton JA, Köttgen A, Bergmann S, Mooser V, Chaturvedi N, Frayling TM, Islam M, Jafar TH, Erdmann J, Kulkarni SR, Bornstein SR, Grässler J, Groop L, Voight BF, Kettunen J, Howard P, Taylor A, Guarrera S, Ricceri F, Emilsson V, Plump A, Barroso I, Khaw KT, Weder AB, Hunt SC, Sun YV, Bergman RN, Collins FS, Bonnycastle LL, Scott LJ, Stringham HM, Peltonen L, Perola M, Vartiainen E, Brand SM, Staessen JA, Wang TJ, Burton PR, Soler Artigas M, Dong Y, Snieder H, Wang X, Zhu H, Lohman KK, Rudock ME, Heckbert SR, Smith NL, Wiggins KL, Doumatey A, Shriner D, Veldre G, Viigimaa M, Kinra S, Prabhakaran D, Tripathy V, Langefeld CD, Rosengren A, Thelle DS, Corsi AM, Singleton A, Forrester T, Hilton G, McKenzie CA, Salako T, Iwai N, Kita Y, Ogihara T, Ohkubo T, Okamura T, Ueshima H, Umemura S, Eyheramendy S, Meitinger T, Wichmann HE, Cho YS, Kim HL, Lee JY, Scott J, Sehmi JS, Zhang W, Hedblad B, Nilsson P, Smith GD, Wong A, Narisu N, Stančáková A, Raffel LJ, Yao J, Kathiresan S, O'Donnell CJ, Schwartz SM, Ikram MA, Longstreth WT Jr, Mosley TH, Seshadri S, Shrine NR, Wain LV, Morken MA, Swift AJ, Laitinen J, Prokopenko I, Zitting P, Cooper JA, Humphries SE, Danesh J, Rasheed A, Goel A, Hamsten A, Watkins H, Bakker SJ, van Gilst WH, Janipalli CS, Mani KR, Yajnik CS, Hofman A, Mattace-Raso FU, Oostra BA, Demirkan A, Isaacs A, Rivadeneira F, Lakatta EG, Orru M, Scuteri A, Ala-Korpela M, Kangas AJ, Lyytikäinen LP, Soininen P, Tukiainen T, Würtz P, Ong RT, Dörr M, Kroemer HK, Völker U, Völzke H, Galan P, Hercberg S, Lathrop M, Zelenika D, Deloukas P, Mangino M, Spector TD, Zhai G, Meschia JF, Nalls MA, Sharma P, Terzic J, Kumar MV, Denniff M, Zukowska-Szczechowska E, Wagenknecht LE, Fowkes FG, Charchar FJ, Schwarz PE, Hayward C, Guo X, Rotimi C, Bots ML, Brand E, Samani NJ, Polasek O, Talmud PJ, Nyberg F, Kuh D, Laan M, Hveem K, Palmer LJ, van der Schouw YT, Casas JP, Mohlke KL, Vineis P, Raitakari O, Ganesh SK, Wong TY, Tai ES, Cooper RS, Laakso M, Rao DC, Harris TB, Morris RW, Dominiczak AF, Kivimaki M, Marmot MG, Miki T, Saleheen D, Chandak GR, Coresh J, Navis G, Salomaa V, Han BG, Zhu X, Kooner JS, Melander O, Ridker PM, Bandinelli S, Gyllensten UB, Wright AF, Wilson JF, Ferrucci L, Farrall M, Tuomilehto J, Pramstaller PP, Elosua R, Soranzo N, Sijbrands EJ, Altshuler D, Loos RJ, Shuldiner AR, Gieger C, Meneton P, Uitterlinden AG, Wareham NJ, Gudnason V, Rotter JI, Rettig R, Uda M, Strachan DP, Witteman JC, Hartikainen AL, Beckmann JS, Boerwinkle E, Vasan RS, Boehnke M, Larson MG, Järvelin MR, Psaty BM, Abecasis GR, Chakravarti A, Elliott P, van Duijn CM, Newton-Cheh C, Levy D, Caulfield MJ, Johnson T.

Supplementary References

s1 Liese AD, Hense HW, Lowel H, et al. (1999) Association of serum uric acid with all-cause and cardiovascular disease mortality and incident myocardial infarction in the MONICA Augsburg cohort. World Health Organization Monitoring Trends and Determinants in Cardiovascular Diseases. Epidemiology 10: 391–397.

s2 Puddu PE, Lanti M, Menotti A, et al. (2001) Serum uric acid for short-term prediction of cardiovascular disease incidence in the Gubbio population study. Acta Cardiol 56: 243–251.

s3 Fang J, Alderman MH, (2000) Serum uric acid and cardiovascular mortality the NHANES I epidemiological follow-up study, 1971–1992. National Health and Nutrition Examination Survey. JAMA 283: 2404–2410.

s4 Medalie JH, Kahn HA, Neufeld HN, Riss E, and Goldbourt U (1973) Five-year myocardial infarction incidence. II. Association of single variables to age and birthplace. J Chronic Dis 26: 325–349.

s5 Moriarity JT, Folsom AR, Iribarren C, Nieto FJ, and Rosamond WD, (2000) Serum uric acid and risk of coronary heart disease: Atherosclerosis Risk in Communities (ARIC) Study. Ann Epidemiol 10: 136–143.

s6 Shah T, Engmann J, Dale C, et al. (2013) Population Genomics of Cardiometabolic Traits: Design of the University College London-London School of Hygiene and Tropical Medicine-Edinburgh-Bristol (UCLEB) Consortium. PLoS ONE 8(8): e71345. doi:10.1371/journal.pone.0071345

s7 Tomita M, Mizuno S, Yamanaka H, et al. (2000) Does hyperuricemia affect mortality? A prospective cohort study of Japanese male workers. J Epidemiol 10: 403–409.

s8 Köttgen A, Albrecht E, Teumer A, et al. (2013).Genome-wide association analyses identify 18 new loci associated with serum urate concentrations. Nature genetics;45(2):145–54

s9 Kolz M, Johnson T, Sanna S, et al. (2009). Meta-analysis of 28,141 individuals identifies common variants within five new loci that influence uric acid concentrations.PLoS Genetics; Jun;5(6):e1000504

s10 Schunkert H, König IR, Kathiresan S, et al. (2011) Large-scale association analysis identifies 13 new susceptibility loci for coronary artery disease. Nat Genet. 43: 333–338

s11 Coronary Artery Disease (C4D) Genetics Consortium (Writing Committee: Peden JF, Hopewell JC, Saleheen D, et al.). (2011). A genome-wide association study in Europeans and South Asians identifies five new loci for coronary artery disease. Nat Genet. 43: 339–344

s12 CARDIoGRAMplusC4D Consortium, Deloukas P, Kanoni S, Willenborg C, et al. (2013). Large-scale association analysis identifies new risk loci for coronary artery disease. Nat Genet 45:25–33

s13 Global Lipids Genetics Consortium 2013. Discovery and refinement of loci associated with lipid levels Nature Genetics 45, 1274–1283.

S14 Morris AP, Voight BF, Teslovich TM, et al. DIAbetes Genetics Replication And Meta-analysis (DIAGRAM) Consortium. (2012) Large-scale association analysis provides insights into the genetic architecture and pathophysiology of type 2 diabetes. Nat Genet. 44:981–90

s15 Dupuis J, Langenberg C, Prokopenko I, et al. (2010) New genetic loci implicated in fasting glucose homeostasis and their impact on type 2 diabetes risk. Nature Genetics. 42. 105-116.

s16 ICBP, Ehret GB, Munroe PB, Rice KM, et al. (2011). Genetic variants in novel pathways influence blood pressure and cardiovascular disease risk. Nature. 478. 103–109.

s17 Speliotes EK, Willer CJ, Berndt SI, et al. (2010). Association analyses of 249,796 individuals reveal 18 new loci associated with body mass index. Nat Genet 42, 937-948.

s18 Wheeler, JG, Juzwishin, DM, Eriksdottir, G, Gudnason, V, and Danesh J, (2005) Serum Uric Acid and Coronary Heart Disease in 9458 Incident Cases and 155084 Controls: Prospective Study and Meta-Analysis. PLoS Med. 2: 236–243.

s19 Yang Q, Köttgen A, Dehghan A, et al. 2010. Multiple Genetic Loci Influence Serum Urate and Their Relationship with Gout and Cardiovascular Disease Risk Factors Circ Cardiovasc Genet. 3(6): 523–530.

s20 ICBP, Ehret GB, Munroe PB, Rice KM, et al. (2011). Genetic variants in novel pathways influence blood pressure and cardiovascular disease risk. Nature. 478. 103–109.
